# The predictive association between the teaching environment and psychological wellbeing: psychological safety, university belonging, and learning engagement

**DOI:** 10.3389/fpsyg.2026.1983869

**Published:** 2026-10-20

**Authors:** Min Jin, Qi Wang

**Affiliations:** 1 School of Educational Sciences, Shangluo University, Shangluo, China; 2 Department of Mental Health, Shangluo Traditional Chinese Medicine Hospital, Shangluo, China

**Keywords:** cross-sectional survey study, learning engagement, psychological safety, psychological wellbeing, serial indirect association, teaching environment, university belonging

## Abstract

**Objective:**

To examine the predictive association between the teaching environment and psychological wellbeing in survey data and to investigate the serial indirect association under a prespecified ordering involving psychological safety, university belonging, and learning engagement.

**Methods:**

A fixed random seed was used to split 815 complete survey questionnaire records into an EFA group (*n* = 408) and a CFA group (*n* = 407), on which principal axis factoring and confirmatory factor analysis were conducted, respectively. After the measurement structure was confirmed, recursive equations and bootstrap intervals were estimated using the full sample.

**Results:**

The background distributions were similar across the two groups. The EFA retained five factors, and the five-factor CFA model showed good fit, χ^2^(485) = 529.551, CFI = 0.994, and RMSEA = 0.015. The total association between the teaching environment and psychological wellbeing was 0.435, and the full serial indirect association was 0.036. Among six competing orders estimated under identical constraints, the theoretically specified order showed the best relative fit, although its absolute fit remained inadequate.

**Conclusions:**

The five variables were interrelated yet distinguishable in the survey data. The proposed ordering may serve as a theoretical model for testing in future studies using authentic samples; the cross-sectional survey findings cannot establish a causal ordering.

## 1 Introduction

The higher education environment not only supports learning activities but also constitutes the everyday context in which students experience teacher support, the peer climate, institutional fairness, and psychological services. In the current era, higher education is undergoing a profound transformation marked by the deep integration of AI technologies. Rather than diminishing the need for human connection, this digital shift makes supportive instructional environments and interpersonal relational climates even more critical for students' psychological experiences (Hu et al., 2026). Students' evaluations of these environmental features may be concurrently associated with psychological safety, university belonging, learning engagement, and psychological wellbeing. Psychological wellbeing refers not merely to short-term pleasant affect or immediate life satisfaction, but to relatively stable positive psychological functioning encompassing self-acceptance, positive relations, autonomy, environmental mastery, purpose in life, and personal growth (Ryff, 1989; Ryff and Keyes, 1995). During higher education, students must simultaneously manage academic demands, relational adjustment, and developmental choices; psychological wellbeing can therefore serve as an important indicator of overall psychological adjustment. Recent literature increasingly highlights the critical importance of studying psychological wellbeing in educational settings, particularly its intrinsic relationships with academic experiences, social support, and students' overall psychological functioning (Mukhlis et al., 2024). In this study, the teaching environment refers to students' overall perceptions of teacher guidance, peer support, psychological services, institutional fairness, and feedback channels, rather than to teaching staff or facilities alone. Previous research has shown that the school environment, university belonging, and learning engagement are interrelated yet distinguishable and that each is statistically associated with psychological safety and psychological wellbeing (Wang and Degol, 2016; Zhang et al., 2025).

Relevant theories and prior research suggest that the teaching environment, psychological safety, university belonging, learning engagement, and psychological wellbeing are typically associated at the same point in time; however, cross-sectional research cannot establish their temporal ordering (Allen et al., 2018; Norabuena-Figueroa et al., 2025). Conceptually, these constructs operate at different levels: psychological safety concerns whether students feel able to voice questions, seek help, and try new approaches (Edmondson, 1999; Frazier et al., 2017); university belonging concerns whether students feel accepted, respected, and included in the school community (Goodenow, 1993; Osterman, 2000); and learning engagement encompasses behavioral, emotional, and cognitive participation (Fredricks et al., 2004). While individual links have been noted in recent literature (Chaudhry et al., 2024), a comprehensive systemic understanding requires addressing four distinct gaps. Conceptually, it remains unclear exactly how environmental factors, relational experiences, learning engagement, and wellbeing are interconnected. Methodologically, a measurement gap exists regarding whether the adapted instruments can adequately distinguish psychological safety, belonging, and engagement. Empirically, integrated evidence is limited among higher education students. Analytically, the proposed sequential ordering among these variables requires clarification.

To systematically address these gaps, the present study adopts Self-Determination Theory (SDT; Deci and Ryan, 2000) as a non-causal framework to provide a strong theoretical justification for our proposed ordering. This approach is highly pertinent, as recent SDT-based structural analyses in Chinese higher education demonstrate that supportive teaching environments—even those integrated with emerging AI technologies—significantly mediate students' learning engagement and psychological flourishing (Chen et al., 2025). We theorize that a supportive teaching environment fulfills needs for autonomy and competence, facilitating psychological safety; this safe climate in turn promotes relatedness, fostering university belonging; these stable psychological and relational resources enable deeper learning engagement, which ultimately manifests in enhanced psychological wellbeing. Following this pre specified sequence, the present study examines the discriminant validity and overall correlational structure of the five variables, estimates their sequential indirect associations, and evaluates robustness through competing orders, background variables, and subgroup analyses.

This study makes four principal contributions. First, it places the teaching environment, psychological safety, university belonging, learning engagement, and eudaimonic psychological wellbeing within a single statistical model. Second, it reports the direct association, seven specific indirect associations, and the full serial indirect association, thereby avoiding the selective presentation of a single pathway. Third, it examines adjustment for demographic variables, differences across background variables, subgroup serial associations, between-subgroup differences, six competing orders under identical constraints, rank transformation, and equal-weighted scoring of psychological wellbeing.

## 2 Theoretical framework and hypotheses

### 2.1 Teaching environment and psychological wellbeing

Anchored primarily in Self-Determination Theory (SDT; Deci and Ryan, 2000), the theoretical framework of this study posits that external educational contexts facilitate positive psychological functioning by fulfilling students' basic psychological needs for autonomy, competence, and relatedness. To avoid an overly eclectic approach, ecological systems theory (Bronfenbrenner, 1979) and specific wellbeing frameworks are integrated merely as complementary perspectives. Research on the school environment further regards instructional support, interpersonal relationships, safety, and the institutional environment as interrelated but distinguishable dimensions (Wang and Degol, 2016; Xu et al., 2024). Accordingly, the present study defines the teaching environment as students' overall perceptions of the learning, relational, psychological, and institutional resources provided by their institution. It is crucial to clarify the conceptual boundary here: the teaching environment represents broad, exogenous educational provisions—such as objective feedback channels, institutional rules, and service availability—which are conceptually distinct from students' subsequent subjective, internal psychological evaluations (e.g., feelings of safety and belonging). Teaching practices that respect student perspectives may therefore accompany experiences of autonomy; constructive feedback may accompany experiences of competence; and relational support may also be associated with the relatedness needs that underpin psychological wellbeing. On this basis, the present study proposes a correlational hypothesis between the teaching environment and psychological wellbeing, without treating the teaching environment as a definitive cause of changes in psychological wellbeing.

H1: The teaching environment is significantly and positively correlated with psychological wellbeing.

### 2.2 Teaching environment and psychological safety

The concept of psychological safety originated in discussions of whether individuals can bring their authentic selves to role-related activities. Kahn (1990) regarded it as a psychological condition in which individuals can express themselves without excessive concern about damage to their image or relationships. While initially conceptualized in organizational literature as a shared team belief (Edmondson, 1999), psychological safety is explicitly operationalized in the present study at the individual level of analysis—capturing an individual student's private perception of whether interpersonal risk-taking is safe, rather than assessing an aggregated group or institutional climate. In teaching settings, psychological safety concerns whether students feel respected when asking questions, acknowledging a lack of understanding, requesting help, or expressing divergent views. Teachers' responses, peers' tolerance of differing perspectives, and the credibility of feedback channels may all be associated with this sense of safety. A stable, respectful, and inclusive teaching environment generally co-occurs with higher psychological safety. Research highlights that encouraging socially responsible institutional leadership is crucial for alleviating students' fears of expression, thereby cementing this psychological safety in higher education settings (Kyambade et al., 2024). However, the association may also be influenced by prior interpersonal experiences, dispositional tendencies, and institutional culture.

H2: The teaching environment is significantly and positively correlated with psychological safety.

### 2.3 Psychological safety and university belonging

Psychological safety and university belonging are adjacent but not interchangeable constructs. Psychological safety addresses the question, “Can I safely speak up, seek help, and make mistakes?” University belonging addresses the question, “Am I accepted by people here, and do I regard myself as a member of the school community?” The former centers on judgments of interpersonal risk in specific interactions, whereas the latter concerns a more enduring sense of membership, emotional connection, and being valued. Belongingness theory proposes that stable, positive, and enduring interpersonal relationships are generally associated with stronger learning motivation and better psychological adjustment (Baumeister and Leary, 1995). Within the overarching SDT framework, the psychological safety to interact without fear logically facilitates the deeper internalization of relatedness needs. University belonging specifically emphasizes whether students feel accepted, respected, and supported by teachers and peers (Goodenow, 1993; Osterman, 2000). Accordingly, the present study treats psychological safety and university belonging as two distinguishable yet potentially positively associated constructs.

H3: Psychological safety is significantly and positively correlated with university belonging.

### 2.4 University belonging and learning engagement

Learning engagement describes students‘ state of participation in learning activities and commonly comprises behavioral, emotional, and cognitive dimensions. Behavioral engagement is reflected in attendance, participation, effort, and persistence; emotional engagement is reflected in interest, valuing, and positive experiences; and cognitive engagement involves strategy use, deep processing, and self-regulation (Fredricks et al., 2004). The development-in-sociocultural-context model of student engagement situates learning engagement within the joint context of individual characteristics and the teaching environment, with both relational experiences and self-appraisals associated with participation in learning (Wang et al., 2019). When needs for relatedness and security are satisfied through a strong sense of university belonging, cognitive and emotional resources are freed and directed toward active behavioral participation. Students with a stronger sense of university belonging also tend to report greater classroom participation and persistence. Recent studies similarly indicate that learning engagement—especially cognitive flow and active participation in modern, blended teaching environments—is intrinsically interrelated with students' overall psychological wellbeing and sense of safety (Shi et al., 2025; Zhu et al., 2024). The present study therefore proposes a positive correlation between university belonging and learning engagement.

H4: University belonging is significantly and positively correlated with learning engagement.

### 2.5 Learning engagement and psychological wellbeing

Learning engagement and psychological wellbeing both have positive connotations, but they operate at different theoretical levels. Learning engagement focuses on the energy, attention, effort, and persistence that students invest in learning activities, whereas psychological wellbeing concerns self-acceptance, positive relations, autonomy, environmental mastery, purpose in life, and personal growth (Ryff, 1989). Students may invest substantial energy over a short period without simultaneously experiencing high psychological wellbeing; conversely, high psychological wellbeing does not imply equally strong engagement in every course. From the perspective of self-determination theory, learning experiences that support autonomy, competence, and relatedness manifest behaviorally as engagement, which is generally associated with a clearer sense of purpose and more positive psychological functioning (Deci and Ryan, 2000; Ryff and Keyes, 1995). This perspective is firmly reinforced by contemporary studies confirming that robust learning engagement serves as a key predictor of university students' overall perceived satisfaction and psychological wellbeing (Nguyen et al., 2026). Accordingly, the present study proposes only that learning engagement and psychological wellbeing are positively correlated.

H5: Learning engagement is significantly and positively correlated with psychological wellbeing.

### 2.6 Hypothesis concerning serial associations

With SDT as the primary explanatory mechanism, the proposed structural associations link these constructs conceptually. In this framework, the teaching environment represents perceptions of the external context and objective resources; psychological safety and university belonging represent the subjective fulfillment of safety and relatedness needs; learning engagement represents a state of participation in learning; and psychological wellbeing represents positive psychological functioning. The equations are therefore ordered as follows: teaching environment–psychological safety–university belonging–learning engagement–psychological wellbeing. Because the data consist of cross-sectional survey records, this ordering is used solely to compare covariance structures and decompose indirect associations; it does not represent an established temporal process (Maxwell and Cole, 2007). Terms such as “pathway” or “serial” herein strictly denote serial indirect associations under a prespecified ordering, rather than an unfolding, temporal psychological mechanism.

H6: The teaching environment, psychological safety, university belonging, learning engagement, and psychological wellbeing form a significant positive serial indirect association under the prespecified ordering. The hypothesized model of associations is illustrated in Figure 1.

**Figure 1:**
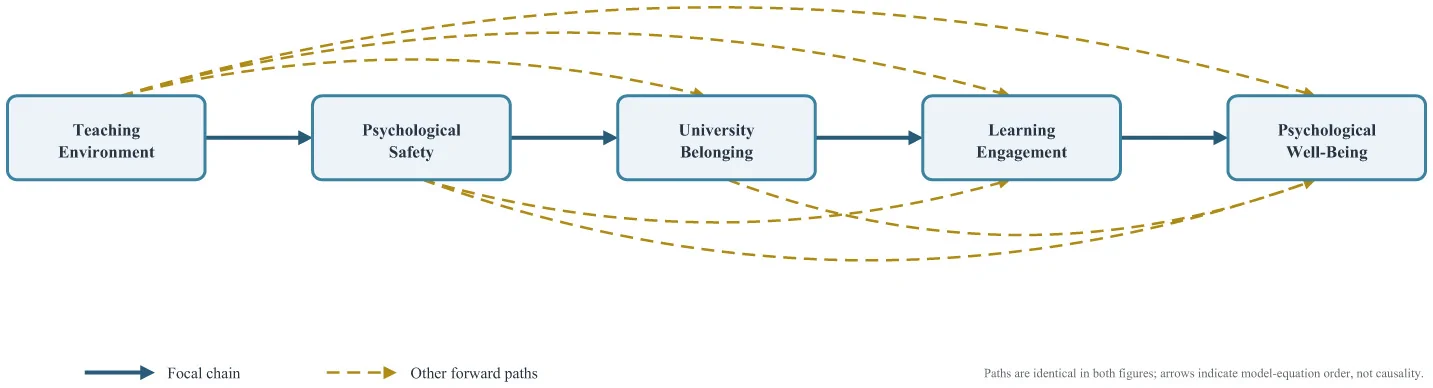
Hypothesized model of associations. The teaching environment, psychological safety, university belonging, learning engagement, and psychological wellbeing are arranged sequentially to test the proposed structural associations. Solid lines denote the adjacent focal chain, and dashed lines denote the other six forward paths. Arrows indicate the sequence of the estimated equations in the cross-sectional design, rather than causal or temporal effects.

## 3 Methods

### 3.1 Design and sample

Data were collected between October and December 2025. The questionnaire was administered in Chinese using Wenjuanxing, a widely adopted professional survey platform in China. Participants were recruited primarily through unversity counselors and course instructors in the targeted cities, who forwarded the survey link to their respective student groups. The survey covered higher education institutions nationwide, including eastern, western, southern, and northern regions. Survey cities were selected using a combination of regional stratification and multistage probability sampling. First, a roster of eligible cities was compiled and each city was assigned a uniform identifier. Second, for each city, the proportion of the national resident population and the proportion of all regular higher education institutions nationwide were calculated. The selection weight was then computed as follows: composite city sampling weight = population share × 50% + share of higher education institutions × 50%. A fixed random seed was set in SPSS 20.0, and four cities were randomly selected from each region according to the composite weights, yielding 16 cities in total. City sample quotas were allocated according to the composite weights, such that city sample size = regional target sample size × the city's composite weight ÷ the sum of the composite weights of selected cities. To prevent excessive sample concentration, no single city was assigned more than 35% of the total sample, that is, 284 respondents. To ensure adequate statistical power for structural equation modeling and detecting small-to-medium indirect associations, an *a priori* sample size calculation was conducted. Based on conventional SEM precision criteria (which recommend a minimum of 200 cases) and G^*^Power estimations for regressions with multiple predictors (assuming a small effect size *f* ^2^= 0.05, α = 0.05, power = 0.95), the minimum required sample size was approximately 400. Thus, the final secured sample of 815 respondents provided highly adequate statistical power and permitted the robust splitting of the sample for independent exploratory and confirmatory factor analyses.

### 3.2 Inclusion and exclusion criteria

The prespecified inclusion criteria for the survey were: (1) aged 18 years or older; (2) currently enrolled at a higher education institution; (3) having read the study information and voluntarily agreed to participate; (4) having completed all 33 core-scale items and the attention-check item; (5) having responses to the core items within the valid range of 1–5; and (6) having a unique record ID. Eligibility, voluntary participation, completeness, and coding ranges should remain traceable in a formal survey (Eysenbach, 2004; von Elm et al., 2007).

The prespecified exclusion criteria were: (1) failure to meet the age or enrollment requirements, or lack of consent to participate; (2) missing, non-numeric, or out-of-range responses to core items; (3) duplicate IDs or completely duplicated rows; (4) failure on the attention check; (5) identical responses to all 33 core items, or long-string responding accompanied by other quality indicators such as attention-check failure, extremely low within-person item variability, or implausible response duration; and (6) a completion time clearly shorter than a reasonable reading time. Invalid responding may take random and non-random forms, and no single indicator reliably identifies all problems. Accordingly, multiple indicators should be evaluated jointly rather than automatically deleting records on the basis of a single variable score or a single long-string index (Curran, 2016; Meade and Craig, 2012). Because the present survey file did not include response duration, criterion (6) could not be applied.

### 3.3 Measures

All five core variables were scored using a 5-point Likert agreement scale, where 1 represented “strongly disagree” and 5 represented “strongly agree”; each variable score was calculated as the mean of its constituent items. To ensure contextual appropriateness for higher education, a rigorous instrument-adaptation process was conducted. This involved translation and back-translation, expert panel reviews for content validity, and cognitive interviews alongside pilot testing with university students to refine item wording. All 33 finalized items are provided in Supplementary Material 3. Throughout this study, the five variables are termed the teaching environment, psychological safety, university belonging, learning engagement, and psychological wellbeing. To maintain consistency with the dataset fields and statistical output, the abbreviations EES, PS, SB, LE, and PWB are retained, respectively.

#### 3.3.1 Teaching environment (EES)

The scale comprises eight items developed with reference to Wang and Degol (2016) dimensions of instructional support, interpersonal relationships, safety, and the institutional environment within the broader school environment. Adapted to higher education, the items assess teacher guidance, peer support, psychological services, institutional fairness, and responsiveness to feedback. These diverse domains were theoretically conceptualized to converge into a unified construct, capturing an overarching institutional learning climate. Higher scores indicate more positive evaluations of the teaching environment.

#### 3.3.2 Psychological safety (PS)

The scale comprises five items based on Edmondson (1999) definition of beliefs about the safety of interpersonal risk taking and informed by the psychological safety framework reviewed by Frazier et al. (2017). Unlike organizational studies that emphasize group-level climate, this scale specifically assesses individual perceptions of safety within the learning context. Content concerning speaking up, asking questions, seeking help, and being permitted to make mistakes was adapted to the teaching context. Higher scores indicate a greater sense that students can safely express themselves and seek help during learning interactions.

#### 3.3.3 University belonging (UB)

The scale comprises five items informed by Goodenow's (1993) Psychological Sense of School Membership scale and Osterman's (2000) framework of interpersonal belonging at school. The item content was contextualized from traditional secondary school settings to specifically reflect interpersonal integration in higher education environments. It measures students' sense of membership, experiences of acceptance, emotional connection, and interpersonal participation. Higher scores indicate a stronger sense of university belonging.

#### 3.3.4 Learning engagement (LE)

The scale comprises six items based on the behavioral, emotional, and cognitive engagement framework proposed by Fredricks et al. (2004). In the context of higher education learning activities, it assesses energy, attention, effort, persistence, classroom participation, and deep understanding. Higher scores indicate greater learning engagement.

#### 3.3.5 Psychological wellbeing (PWB)

The scale comprises nine items based on the eudaimonic psychological wellbeing framework of Ryff (1989) and Ryff and Keyes (1995), covering six domains: self-acceptance, positive relations, autonomy, environmental mastery, purpose in life, and personal growth. Given the limited number of items per domain, this measure was operationalized as a brief overall PWB index informed by Ryff's framework, rather than a full multidimensional subscale representation; thus, only the overall mean score was calculated. Higher scores indicate greater overall psychological wellbeing.

The structures of these scales were first explored in the randomly selected EFA group and then confirmed in the independent CFA group. Reliability, convergent validity, and discriminant validity are reported for the CFA group. The measurement structure and item assignments were established before the full-sample path analysis and were not modified on the basis of path significance.

### 3.4 Analytical procedure and methods

The statistical procedure comprised the following steps: (1) verifying data quality and sample flow; (2) randomly splitting the sample using a fixed seed and testing the comparability of the two groups; (3) exploring the factor structure in the EFA group; (4) testing competing measurement models, reliability, and validity in the independent CFA group; (5) testing measurement invariance to ensure valid multigroup comparisons; (6) after confirming the structure, estimating paths, effect sizes, and bootstrap indirect associations in the full sample; (7) comparing six competing sequential orderings based on identical covariance structures; and (8) incorporating specific background variables as literature-supported control covariates and conducting subgroup robustness analyses.

To avoid exploring and confirming the factor structure in the same cases, the unique ID was used as the sampling unit. A NumPy PCG64 generator and a fixed seed of 20260826 were used to produce a single random permutation: the first 408 records were assigned to the EFA group and the remaining 407 to the CFA group, with no overlap. The split was determined before factor analysis; neither the seed nor the draw was repeated in response to the balance-test results. Separating exploration from confirmation can reduce the optimistic bias associated with repeatedly fitting a model to the same sample (Fokkema and Greiff, 2017; Worthington and Whittaker, 2006).

Principal axis factoring with Promax oblique rotation was conducted on the 33 core items in the EFA group. The number of factors was determined primarily from the 95th-percentile random eigenvalues generated in 1,000 parallel-analysis replications (seed = 20260827). KMO, Bartlett's test of sphericity, the number of factors with eigenvalues greater than 1, primary loadings, cross-loadings, communalities, and factor correlations were also reported. Loadings with absolute values below 0.20 were omitted from the table.

Maximum-likelihood estimation was used in the CFA group to compare a five-factor model, two four-factor models, a three-factor model, and a one-factor model. The reported indices were χ^2^, χ^2^/df, CFI, TLI, RMSEA, and SRMR, and the findings were interpreted against commonly used reference values (Hu and Bentler, 1999). Internal consistency was assessed using Cronbach's α and composite reliability (CR), convergent validity using AVE, and discriminant validity using both the Fornell–Larcker criterion and HTMT < 0.85 (Fornell and Larcker, 1981; Henseler et al., 2015). Harman's single-factor test and the forced one-factor model were used only as diagnostics for common method concerns and were not regarded as sufficient to rule out common method bias (Podsakoff et al., 2003).

Before conducting subgroup comparisons, measurement invariance testing (configural, metric, and scalar invariance) was performed using multigroup CFA to establish that the observed subgroup similarities or differences reflect genuine structural associations rather than differential item functioning. After the measurement structure had been confirmed in the independent sample, all 815 records were used for recursive regression, bootstrap, subgroup, and robustness analyses to preserve the precision of path estimates. Item assignments were not modified using the full sample at this stage. The recursive model successively treated PS, SB, LE, and PWB as outcome variables and simultaneously included all variables theoretically preceding each outcome. The total association between EES and PWB was estimated separately.

All core variables were standardized before analysis. Standardized regression coefficients, HC3 heteroskedasticity-robust standard errors, *t* values, *p* values, 95% confidence intervals, *R*^2^, and *f*^2^ are reported. The *f*^2^ values were calculated from the change in *R*^2^ after deleting each predictor and were interpreted as small, medium, and large at 0.02, 0.15, and 0.35, respectively (Cohen, 1988; Long and Ervin, 2000). Percentile 95% confidence intervals for indirect associations were obtained from 5,000 bootstrap resamples. These estimates are presented as coefficient decompositions under the prespecified equation ordering, rather than as estimates of causal mediation (Hayes, 2022; Preacher and Hayes, 2008; Zhao et al., 2010).

The competing-order analysis treated the six permutations of PS, SB, and LE as candidate orders. To ensure comparability, all models used the same five composite mean scores and the same constraints: EES was connected to the first intermediate variable, the three intermediate variables were connected sequentially, and both the final intermediate variable and EES entered the PWB equation. The models had the same number of parameters and were estimated by maximum likelihood. Reported indices were χ^2^, df, χ^2^/df, CFI, TLI, RMSEA, SRMR, AIC, and BIC. The full serial indirect association was estimated using 5,000 bootstrap resamples, with the estimate, bootstrap SE, percentile 95% CI, and empirical two-sided *p* value reported. This analysis compares only the relative compatibility of cross-sectional covariance structures and is not used to establish a temporal or causal ordering.

Background variables, including specific demographic and academic characteristics, were selected based on existing literature suggesting their potential confounding effects on wellbeing and engagement. Thus, these variables were controlled for in extended sensitivity path analyses. Robustness analyses included adjustment for demographic variables A1–A5, adjustment for the extended set of variables A1–A8, equal-weighted scoring of the six PWB domains, rank transformation, bootstrapping with a second random seed, and exclusion of the case flagged for data quality.

In addition, for tests of random-group comparability, background-variable differences in mean PWB, and the 28 cross-tabulations of background variables, the categories 24–26 years and 27 years or older were combined as “24 years or older,” and master's and doctoral students were combined as “graduate students”; all other categories were retained. Pearson's χ^2^ was used for the cross-tabulations, and expected frequencies were examined. The merging rules were applied consistently on the basis of the substantive similarity of adjacent categories and the risk of sparse cells, rather than selected according to statistical significance (Cochran, 1954). The subsequent cross-tabulations in the results continue to report the original four-category classifications.

## 4 Results

### 4.1 Sample characteristics

The characteristics of the survey sample are presented in Table 1.

**Table 1:** Sample characteristics (*N* = 815).

Variable	Category	*n*	%
Gender	Male	355	43.6%
	Female	460	56.4%
Age	18–20 years	426	52.3%
	21–23 years	277	34.0%
	24–26 years	90	11.0%
	27 years or older	22	2.7%
Level of study	Junior college	81	9.9%
	Undergraduate	595	73.0%
	Master's student	118	14.5%
	Doctoral student	21	2.6%
Academic field	Humanities and Social Sciences	306	37.5%
	Science and Engineering	273	33.5%
	Medicine or Agriculture	148	18.2%
	Arts or Sports	88	10.8%
Living arrangement	On-campus residence	372	45.6%
	Off-campus rental accommodation	190	23.3%
	Living with family	198	24.3%
	Other	55	6.7%
Self-rated academic performance	Poor	190	23.3%
	Average	231	28.3%
	Good	234	28.7%
	Excellent	160	19.6%
Academic stress	Very low	172	21.1%
	Low	258	31.7%
	High	237	29.1%
	Very high	148	18.2%
Frequency of psychological distress	Never	172	21.1%
	Occasionally	279	34.2%
	Often	235	28.8%
	Almost always	129	15.8%

All 33 core items fell within the valid range of 1–5, and no severe univariate skewness was observed. The mean scores of the five variables matched, item by item, the values recalculated from the raw items, with a maximum absolute difference of 0.000. Applying the prespecified rules yielded an EFA group of 408 records and a CFA group of 407 records, with no overlapping IDs. After sparse categories for age and level of study were merged consistently, none of the eight tests of background-variable distributions between groups was significant (*p* = 0.173–0.918); Cramér's V ranged from 0.011 to 0.078, indicating no substantively meaningful imbalance (Table 2).

**Table 2:** Comparability of the randomly split groups.

Variable	EFA group (*n* = 408)	CFA group (*n* = 407)	χ^2^	*df*	*p*	*V*
Gender	Male, 180 (44.1%); female, 228 (55.9%)	Male, 175 (43.0%); female, 232 (57.0%)	0.104	1	0.747	0.011
Age	18–20 years, 223 (54.7%); 21–23 years, 131 (32.1%); 24 years or older, 54 (13.2%)	18–20 years, 203 (49.9%); 21–23 years, 146 (35.9%); 24 years or older, 58 (14.3%)	1.893	2	0.388	0.048
Level of study	Junior college, 43 (10.5%); undergraduate, 301 (73.8%); graduate, 64 (15.7%)	Junior college, 38 (9.3%); undergraduate, 294 (72.2%); graduate, 75 (18.4%)	1.260	2	0.533	0.039
Academic field	Humanities and Social Sciences, 152 (37.3%); Science and Engineering, 136 (33.3%); Medicine or Agriculture, 78 (19.1%); Arts or Sports, 42 (10.3%)	Humanities and Social Sciences, 154 (37.8%); Science and Engineering, 137 (33.7%); Medicine or Agriculture, 70 (17.2%); Arts or Sports, 46 (11.3%)	0.630	3	0.890	0.028
Living arrangement	On-campus residence, 187 (45.8%); off-campus rental accommodation, 99 (24.3%); living with family, 98 (24.0%); other, 24 (5.9%)	On-campus residence, 185 (45.5%); off-campus rental accommodation, 91 (22.4%); living with family, 100 (24.6%); other, 31 (7.6%)	1.257	3	0.739	0.039
Academic performance	Poor, 93 (22.8%); average, 115 (28.2%); good, 116 (28.4%); excellent, 84 (20.6%)	Poor, 97 (23.8%); average, 116 (28.5%); good, 118 (29.0%); excellent, 76 (18.7%)	0.504	3	0.918	0.025
Academic stress	Very low, 78 (19.1%); low, 130 (31.9%); high, 115 (28.2%); very high, 85 (20.8%)	Very low, 94 (23.1%); low, 128 (31.4%); high, 122 (30.0%); very high, 63 (15.5%)	4.980	3	0.173	0.078
Frequency of psychological distress	Never, 90 (22.1%); occasionally, 132 (32.4%); often, 121 (29.7%); almost always, 65 (15.9%)	Never, 82 (20.1%); occasionally, 147 (36.1%); often, 114 (28.0%); almost always, 64 (15.7%)	1.394	3	0.707	0.041

Cells show *n* (within-group %). Age was grouped as 18–20 years, 21–23 years, and 24 years or older; level of study was grouped as junior college, undergraduate, and graduate (master's and doctoral students combined). Group assignment was based on a single permutation generated using the fixed random seed 20260826; p is the two-sided value from Pearson's chi-square test. All expected frequencies exceeded 5. Detailed demographic cross-tabulations and chi-square results are provided in Supplementary Material 2.

### 4.2 Exploratory factor analysis

In the EFA group, the KMO value was 0.949 and Bartlett's test of sphericity was significant, χ^2^ (528) = 8258.943, *p* < 0.001. Parallel analysis retained five factors. After Promax rotation, every item had its highest loading on the theoretically assigned factor. Primary loadings ranged from 0.646 to 0.844, the largest cross-loading was 0.153, communalities ranged from 0.532 to 0.720, and the five-factor solution explained 59.48% of the common variance. The factor pattern was consistent with the prespecified structures of EES, PS, SB, LE, and PWB (Tables 3, 4). To provide comprehensive item-level transparency for the adapted instruments, the complete factor matrix—detailing all factor loadings, cross-loadings, and communalities—is presented in Supplementary Table S1.

**Table 3:** Rotated EFA loadings (*N* = 408).

Variable	Number of items	Range of primary loadings	Range of communalities
Teaching environment (EES)	8	0.734~0.786	0.545~0.652
Psychological safety (PS)	5	0.706~0.844	0.538~0.720
University belonging (SB)	5	0.724~0.835	0.569~0.631
Learning engagement (LE)	6	0.647~0.828	0.537~0.657
Psychological wellbeing (PWB)	9	0.646~0.818	0.532~0.634

Extraction was performed using principal axis factoring with Promax rotation.

**Table 4:** EFA suitability and structure (*N* = 408).

Indicator	Result	Interpretation
KMO	0.949 (lowest item value = 0.925)	Suitable for factor analysis
Bartlett's Test of Sphericity	χ^2^ = 8258.943, *df* = 528, *p* < 0.001	The correlation matrix is not an identity matrix
Parallel analysis	Five factors retained; fifth eigenvalue, 1.424 > 1.391; sixth eigenvalue, 0.712 < 1.347	Supports the five-factor structure
Eigenvalues > 1	Five factors	Consistent with parallel analysis
Common variance explained	59.48%	Five-factor principal axis solution
Primary loadings	0.646−0.844	All ≥ 0.40
Largest cross-loading	0.153	Below 0.20
Communalities	0.532−0.720	All ≥ 0.40
Factor correlations	0.364−0.616	Correlated but distinguishable

Parallel analysis was based on the 95th-percentile eigenvalues from 1,000 random correlation matrices of the same dimensions (seed = 20260827). The percentage of common variance explained was based on the five-factor principal axis solution.

### 4.3 Confirmatory measurement analysis

In the independent CFA group, α and CR exceeded 0.88 for all five variables, AVE exceeded 0.50 for each variable, and standardized loadings ranged from 0.678 to 0.838. The diagonal Fornell–Larcker values exceeded the corresponding interconstruct correlations, and the maximum HTMT value was 0.617, supporting convergent and discriminant validity (Tables 5, 6). Complete item-level standardized factor loadings are provided in Supplementary Table S2.

**Table 5:** CFA reliability and convergent validity (*N* = 407).

Variable	*k*	α	CR	AVE	Loading range	CITC range
EES	8	0.926	0.927	0.614	0.704–0.833	0.676–0.793
PS	5	0.895	0.895	0.631	0.749–0.838	0.700–0.779
SB	5	0.882	0.884	0.604	0.702–0.827	0.654–0.770
LE	6	0.906	0.906	0.618	0.750–0.829	0.705–0.778
PWB	9	0.907	0.907	0.521	0.678–0.756	0.640–0.720

α and CITC were calculated from the raw item responses in the CFA group; CR, AVE, and the loading ranges were derived from the standardized solution of the maximum-likelihood five-factor CFA. PWB is reported as an overall nine-item short form.

**Table 6:** CFA correlations, HTMT, and square roots of AVE (*N* = 407).

Variable	EES	PS	SB	LE	PWB
EES	0.783	0.599	0.449	0.359	0.476
PS	0.546	0.794	0.588	0.492	0.493
SB	0.406	0.522	0.777	0.617	0.520
LE	0.330	0.444	0.553	0.786	0.616
PWB	0.436	0.443	0.465	0.558	0.722

The lower triangle shows Pearson correlations among variable means, the upper triangle shows HTMT, and the diagonal shows the square root of AVE. All Pearson correlations were significant at *p* < 0.001; the maximum HTMT was 0.617.

In the independent CFA group drawn from the survey data, the five-factor model showed good fit under maximum-likelihood estimation, χ^2^(485) = 529.551, χ^2^/*df* = 1.092, CFI = 0.994, TLI = 0.994, RMSEA = 0.015, and SRMR = 0.031, and clearly outperformed the two four-factor models, the three-factor model, and the one-factor model (Table 7). Because the pre specified structure was supported in the holdout sample, no correlated item residuals were added and no variable assignments were changed on the basis of modification indices. While these CFA results provide profound evidence for the internal structure of the measures within this specific dataset, they serve as a complement to, rather than a definitive substitute for, comprehensive construct validity.

**Table 7:** Comparison of CFA measurement models (*N* = 407).

Model	χ^2^	*df*	χ^2^/*df*	CFI	TLI	RMSEA	SRMR
Five-factor	529.551	485	1.092	0.994	0.994	0.015	0.031
Four-factor A (PS + SB)	1,119.530	489	2.289	0.921	0.915	0.056	0.053
Four-factor B (SB + LE)	1,089.721	489	2.228	0.925	0.919	0.055	0.054
Three-factor (PS + SB + LE)	1,802.342	492	3.663	0.836	0.824	0.081	0.075
One-factor	3,801.114	495	7.679	0.585	0.558	0.128	0.118

Four-factor model A combined PS and SB; four-factor model B combined SB and LE; and the three-factor model combined PS, SB, and LE. All models used the same 33 items and maximum-likelihood estimation.

In the CFA group, the diagnostic one-factor model for the nine PWB items yielded χ^2^(27) = 90.940, χ^2^/*df* = 3.368, CFI = 0.964, TLI = 0.952, RMSEA = 0.076, and SRMR = 0.034, meeting commonly used overall fit criteria. Because each content domain was represented by only one or two items, this result supports only the use of an overall short form and does not establish separate measurement of the six domains.

### 4.4 Measurement invariance testing

Before examining subgroup differences in subsequent analyses, measurement invariance testing (configural, metric, and scalar) was conducted across key demographic subgroups using multigroup CFA. The configural model established a baseline fit (χ^2^ = 1,232.827, *df* = 970, CFI = 0.984, RMSEA = 0.026). Subsequent metric and scalar invariance models yielded ΔCFI ≤ 0.01 and ΔRMSEA ≤ 0.015, establishing strong measurement equality. This invariance evidence ensures that observed subgroup similarities or differences in the structural associations reflect genuine psychological variations rather than differential item functioning.

### 4.5 Descriptive statistics of the variables

Descriptive statistics for the variables are presented in Table 8.

**Table 8:** Descriptive statistics of the variables (*N* = 815).

Variable	M	SD	Minimum	Maximum	Skewness	Kurtosis
EES	2.985	0.935	1.000	5.000	0.033	−0.775
PS	2.989	0.961	1.000	5.000	0.067	−0.708
SB	2.998	0.953	1.000	5.000	0.037	−0.673
LE	3.004	0.950	1.000	5.000	−0.017	−0.797
PWB	2.999	0.886	1.000	5.000	0.018	−0.647

The means of all five variables were close to the scale midpoint of 3.000. As hypotheses H1–H5 were formulated as correlational propositions addressing the concurrent interrelatedness of the constructs, they were tested using bivariate correlation estimates. Pearson correlations among the variables ranged from 0.335 to 0.561; all were positive and significant at *p* < 0.001, with their 95% confidence intervals strictly excluding zero (the complete correlation matrix including 95% CIs is provided in Supplementary Table S3). Based on these unadjusted bivariate estimates, theoretical hypotheses H1 (EES with PWB), H2 (EES with PS), H3 (PS with SB), H4 (SB with LE), and H5 (LE with PWB) were fundamentally supported.

### 4.6 Serial path estimates

As detailed in Table 9 and Figure 2, the teaching environment had a relatively strong positive conditional association with psychological safety (β = 0.503, *p* < 0.001). In the subsequent equations, the conditional associations were comparatively strong between PS and SB (β = 0.397), SB and LE (β = 0.455), and LE and PWB (β = 0.395). The unique coefficients for EES with LE and for SB with PWB were small, with f^2^ values of 0.005 and 0.006, respectively. The PWB equation had an R^2^ of 0.390, and the maximum VIF was 1.719, indicating no severe multicollinearity.

**Table 9:** Serial path estimates (*N* = 815).

Outcome variable	Predictor	β	HC3 *SE*	*t*	*p*	95% CI	*R* ^2^	*f*^2^ (Interpretation)
PS	EES	0.503	0.030	16.631	< 0.001	[0.444, 0.563]	0.253	0.339 (medium)
SB	EES	0.212	0.035	6.103	< 0.001	[0.144, 0.281]	0.288	0.047 (small)
	PS	0.397	0.036	10.930	< 0.001	[0.326, 0.468]	0.288	0.165 (medium)
LE	EES	0.070	0.033	2.145	=0.032	[0.006, 0.134]	0.343	0.005 (negligible)
	PS	0.153	0.035	4.343	< 0.001	[0.084, 0.222]	0.343	0.023 (small)
	SB	0.455	0.032	14.166	< 0.001	[0.392, 0.518]	0.343	0.225 (medium)
PWB	EES	0.221	0.031	7.109	< 0.001	[0.160, 0.282]	0.390	0.057 (small)
	PS	0.099	0.035	2.801	=0.005	[0.030, 0.168]	0.390	0.010 (negligible)
	SB	0.077	0.037	2.077	=0.038	[0.004, 0.150]	0.390	0.006 (negligible)
	LE	0.395	0.034	11.687	< 0.001	[0.329, 0.462]	0.390	0.168 (medium)
PWB (Total association)	EES	0.435	0.030	14.353	< 0.001	[0.376, 0.495]	0.189	0.234 (medium)

After the measurement structure was confirmed through independent EFA and CFA, the path model was estimated using the full sample. β denotes the standardized coefficient; confidence intervals, *t* values, and p values are based on HC3 heteroskedasticity-robust standard errors. *R*^2^ is repeated for rows belonging to the same outcome equation. The *f* ^2^ values were interpreted as small, medium, and large at 0.02, 0.15, and 0.35, respectively.

**Figure 2:**
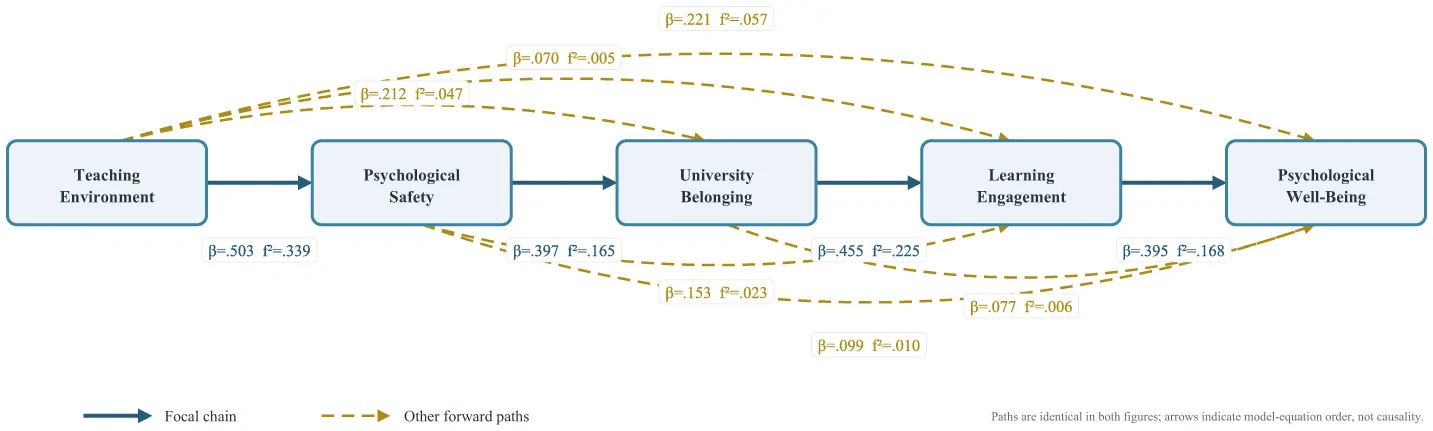
Estimated model of associations. β denotes the standardized regression coefficient, and *f* ^2^ denotes the incremental effect size of the corresponding predictor. Solid lines indicate the adjacent focal chain, and dashed lines indicate the other forward paths. Arrows indicate the sequence of the estimated equations in the cross-sectional design, rather than causal or temporal effects.

### 4.7 Decomposition of cross-sectional associations

As summarized in Table 10, the total predictive association between the teaching environment and PWB was 0.435, the direct association was 0.221, and the total indirect association was 0.214. The full serial indirect association under this prespecified ordering was 0.036, 95% CI [0.026, 0.047]. The bootstrap intervals for all seven specific indirect associations excluded 0, indicating that the data were compatible not only with the full serial chain but also with multiple shorter pathways. Although the standardized estimate of the full sequential mechanism (0.036) appears statistically smaller than the direct paths, such modest specific effect sizes are common and theoretically expected in distal multi-step associational chains within complex educational ecosystems, underscoring the additive rather than absolute nature of these intermediate variables.

**Table 10:** Decomposition of indirect associations (5,000 Bootstrap resamples).

Association/path	Estimate	Boot SE	Boot LLCI	Boot ULCI	Includes 0	Percentage of total association
EES → PS → PWB	0.050	0.018	0.016	0.085	No	11.44%
EES → SB → PWB	0.016	0.008	0.001	0.034	No	3.75%
EES → LE → PWB	0.028	0.013	0.002	0.054	No	6.37%
EES → PS → SB → PWB	0.015	0.008	0.001	0.031	No	3.53%
EES → PS → LE → PWB	0.030	0.007	0.016	0.046	No	7.01%
EES → SB → LE → PWB	0.038	0.007	0.024	0.053	No	8.78%
EES → PS → SB → LE → PWB	0.036	0.005	0.026	0.047	No	8.26%
Total indirect association	0.214	0.022	0.171	0.258	No	49.15%
Direct association	0.221	0.030	0.161	0.281	No	50.85%
Total association	0.435	0.028	0.379	0.489	No	100.00%

The seven specific indirect paths provide a supplementary decomposition of the overall model and do not correspond individually to the study hypotheses. Percentages use the total association of 0.435 as the denominator and are presented only to describe the coefficient decomposition; they should not be interpreted as proportions mediated causally.

### 4.8 Competing-path analysis

Beyond the complete path decomposition in the primary model, the six possible orderings of PS, SB, and LE were tested under identical constraints. The comparison held the observed variables, sample size, degrees of freedom, and number of parameters constant; therefore, differences in fit arose solely from the ordering of the intermediate variables (Table 11).

**Table 11:** Comparison of competing-path model fit.

Order of intermediate variables	χ^2^(*df*)/χ^2^/*df*	CFI/TLI	RMSEA/SRMR	AIC	BIC
PS → SB → LE (theoretical)	88.7(5)/17.7	0.933/0.866	0.143/0.111	10,413.4	10,460.4
PS → LE → SB	298.8(5)/59.8	0.765/0.529	0.269/0.180	10,623.7	10,670.8
SB → PS → LE	326.8(5)/65.4	0.742/0.484	0.281/0.192	10,651.7	10,698.8
LE → SB → PS	391.2(5)/78.2	0.691/0.381	0.308/0.216	10,716.2	10,763.3
SB → LE → PS	418.7(5)/83.7	0.669/0.337	0.319/0.219	10,743.7	10,790.8
LE → PS → SB	509.4(5)/101.9	0.596/0.192	0.352/0.243	10,834.6	10,881.6

*N* = 815. “χ^2^(*df* )/χ^2^/*df* ” reports the chi-square value, degrees of freedom, and relative chi-square, respectively. χ^2^ and χ^2^/*df* are reported to one decimal place; CFI, TLI, RMSEA, and SRMR to three decimal places; and AIC and BIC to one decimal place. The χ^2^ test was significant at *p* < 0.001 for all six models. The models used the same observed variables, constraints, and number of parameters. Lower AIC and BIC values indicate lower relative information loss but do not replace tests of absolute model fit.

As summarized in Table 12, across the six competing orders, the full-chain product terms ranged from 0.022 to 0.065, bootstrap SEs ranged from 0.005 to 0.008, all 95% bootstrap intervals excluded 0, and all empirical two-sided *p* values were below 0.001. The presence of non-zero product terms for multiple orderings indicates that statistical significance of a product term cannot by itself identify the causal sequence of the intermediate variables; judgments about ordering must also draw on model fit, theory, and longitudinal evidence.

**Table 12:** Effects for the competing serial chains.

Order of intermediate variables	Estimate	Boot SE	Boot 95% CI	*p*	Interpretation
PS → SB → LE (theoretical)	0.065	0.008	[0.051, 0.082]	< 0.001	Positive; interval excludes 0
PS → LE → SB	0.037	0.006	[0.026, 0.051]	< 0.001	Positive; interval excludes 0
SB → PS → LE	0.040	0.007	[0.028, 0.054]	< 0.001	Positive; interval excludes 0
LE → SB → PS	0.025	0.005	[0.016, 0.036]	< 0.001	Positive; interval excludes 0
SB → LE → PS	0.025	0.005	[0.016, 0.037]	< 0.001	Positive; interval excludes 0
LE → PS → SB	0.022	0.005	[0.014, 0.032]	< 0.001	Positive; interval excludes 0

The estimate is the product term for the full chain in each constrained model. Bootstrap SE, bootstrap 95% CI, and *p* were based on 5,000 bootstrap resamples.

The theoretical order PS → SB → LE produced the lowest BIC among the six candidate models, with ΔBIC = 210.352 relative to the next-best model; its CFI of 0.933 was also higher than those of the other candidates. Nevertheless, the model had χ^2^/*df* = 17.741, TLI = 0.866, RMSEA = 0.143, and SRMR = 0.111, failing to meet commonly used criteria for absolute fit. Thus, the theoretical order can only be regarded as relatively more compatible with the covariance structure of the survey data; the alternative orders cannot be ruled out by cross-sectional metrics alone.

### 4.9 Differences across background variables

Baseline differences in mean PWB across background variables are summarized in Table 13. After the A1–A5 demographic and educational background variables were added to each path equation, the total association between EES and PWB was 0.429, and the full serial indirect association was 0.035, 95% CI [0.025, 0.046]. After adjustment for A6–A8, the total association was 0.366 and the full serial indirect association was 0.022, 95% CI [0.016, 0.030]; however, the weak EES → LE and SB → PWB paths no longer reached the 0.05 significance level. Because A6–A8 were measured concurrently with psychological wellbeing and may lie within adjacent psychological processes, the extended model is presented only as a conservative sensitivity analysis.

**Table 13:** Background variables and differences in PWB.

Variable	Mean PWB by category	*F*	*p*	η^2^
Gender	Male: 2.944; female: 3.041	2.411	= 0.121	0.003
Age	18–20 years: 2.965; 21–23 years: 3.051; 24 years or older: 3.000	0.798	= 0.450	0.002
Level of study	Junior college: 2.974; undergraduate: 3.028; graduate: 2.887	1.462	= 0.232	0.004
Academic field	Humanities and Social Sciences: 2.925; Science and Engineering: 3.068; Medicine or Agriculture: 2.993; Arts or Sports: 3.049	1.371	= 0.250	0.005
Living arrangement	On-campus residence: 3.075; off-campus rental accommodation: 2.886; living with family: 2.962; other: 3.006	2.057	= 0.104	0.008
Self-rated academic performance	Poor: 2.713; average: 2.903; good: 3.048; excellent: 3.404	20.238	< 0.001	0.070
Academic stress	Very low: 3.165; low: 3.102; high: 2.934; very high: 2.728	8.434	< 0.001	0.030
Frequency of psychological distress	Never: 3.395; occasionally: 3.147; often: 2.828; almost always: 2.463	37.053	< 0.001	0.121

η^2^ is the effect size from one-way analysis of variance. Age was categorized as 18–20 years, 21–23 years, and 24 years or older; level of study was categorized as junior college, undergraduate, and graduate (master's and doctoral students combined). Differences by gender, age, level of study, academic field, and living arrangement did not reach the 0.05 significance level. Concurrent mean differences were observed for self-rated academic performance, academic stress, and frequency of psychological distress.

### 4.10 Subgroup analyses

As detailed in Table 14, point estimates of the full serial association ranged from 0.025 to 0.047 across the 16 subgroups, and all within-group bootstrap intervals excluded 0. However, all eight between-group difference intervals included 0 (Table 15), with *p* = 0.190–0.946. Coupled with the earlier established measurement invariance, these findings suggest that the sequential structural associations operate with remarkable consistency across diverse student demographics and academic profiles.

**Table 14:** Serial Associations by Subgroup.

Grouping variable	Subgroup	*n*	Estimate	Boot SE	Boot 95% CI	Interpretation
Gender	Male	355	0.035	0.009	[0.020, 0.054]	Interval excludes 0
	Female	460	0.034	0.007	[0.022, 0.048]	Interval excludes 0
Age	18–20 years	426	0.036	0.008	[0.023, 0.053]	Interval excludes 0
	21 years or older	389	0.035	0.008	[0.021, 0.050]	Interval excludes 0
Level of study	Junior college/undergraduate	676	0.033	0.005	[0.024, 0.045]	Interval excludes 0
	Graduate	139	0.047	0.018	[0.017, 0.086]	Interval excludes 0
Academic field	Humanities and Social Sciences	306	0.037	0.009	[0.022, 0.058]	Interval excludes 0
	Other academic fields	509	0.035	0.007	[0.023, 0.049]	Interval excludes 0
Living arrangement	On-campus residence	372	0.038	0.008	[0.024, 0.054]	Interval excludes 0
	Other living arrangements	443	0.034	0.007	[0.021, 0.050]	Interval excludes 0
Academic performance	Poor/average	421	0.027	0.006	[0.016, 0.040]	Interval excludes 0
	Good/excellent	394	0.034	0.008	[0.021, 0.051]	Interval excludes 0
Academic stress	Very low/low	430	0.035	0.007	[0.022, 0.051]	Interval excludes 0
	High/very high	385	0.035	0.008	[0.021, 0.051]	Interval excludes 0
Frequency of psychological distress	Never/occasionally	451	0.038	0.008	[0.024, 0.054]	Interval excludes 0
	Often/almost always	364	0.025	0.006	[0.014, 0.039]	Interval excludes 0

Within each subgroup, the variables were restandardized and the full EES → PS → SB → LE → PWB serial association was estimated using 5,000 bootstrap resamples.

**Table 15:** Between-subgroup differences.

Grouping variable	A–B	Δ Estimate	Boot SE	Boot 95% CI	*p*	Inference
Gender	Male—female	0.001	0.011	[−0.020, 0.023]	= 0.922	Difference not significant
Age	18–20 years−21 years or older	0.002	0.011	[−0.018, 0.023]	= 0.869	Difference not significant
Level of study	Junior college/undergraduate—graduate	−0.013	0.018	[−0.054, 0.019]	= 0.517	Difference not significant
Academic field	Humanities and social sciences—other academic fields	0.002	0.011	[−0.019, 0.027]	= 0.856	Difference not significant
Living arrangement	On-campus residence—other living arrangements	0.004	0.011	[−0.018, 0.024]	= 0.758	Difference not significant
Academic performance	Poor/average—good/excellent	−0.007	0.010	[−0.027, 0.012]	= 0.448	Difference not significant
Academic stress	Very low/low —high/very high	0.000	0.010	[−0.020, 0.021]	= 0.946	Difference not significant
Frequency of psychological distress	Never/occasionally— often/almost always	0.012	0.010	[−0.007, 0.032]	= 0.190	Difference not significant

Differences were estimated from the bootstrap distributions of two independently resampled groups. The confidence intervals for all eight differences included 0; accordingly, the results do not support significant differences in the serial association across subgroups defined by gender, age, level of study, academic field, living arrangement, academic performance, academic stress, or frequency of psychological distress.

### 4.11 Robustness checks

The results of the robustness analyses are presented in Table 16.

**Table 16:** Robustness checks.

Analytical specification	Total association	Direct association	Total indirect association (95% CI)	Full serial association (95% CI)	Outcome *R*^2^	Interpretation
Primary model	0.435	0.221	0.214 [0.171, 0.258]	0.036 [0.026, 0.047]	0.390	Stable
Adjusted for A1–A5	0.429	0.220	0.208 [0.165, 0.253]	0.035 [0.025, 0.046]	0.400	Stable
Adjusted for A1–A8	0.366	0.207	0.159 [0.119, 0.198]	0.022 [0.016, 0.030]	0.437	Stable
Equal-weighted six-domain PWB score	0.438	0.221	0.217 [0.173, 0.260]	0.037 [0.027, 0.048]	0.401	Stable
Rank-transformed model	0.416	0.213	0.202 [0.160, 0.247]	0.033 [0.024, 0.043]	0.369	Stable
ID 713 excluded	0.434	0.221	0.212 [0.168, 0.256]	0.036 [0.026, 0.046]	0.389	Stable

All specifications used 5,000 bootstrap resamples. The competing-path comparison is reported separately and is not combined with robustness specifications involving scoring, covariate adjustment, or case handling.

After adjustment for A1–A5, adjustment for A1–A8, equal-weighted PWB scoring, rank transformation, and exclusion of the case flagged for data quality, the full serial association ranged from 0.022 to 0.037, and all bootstrap intervals excluded 0. The effect was smallest in the extended adjustment model, indicating that the weak paths were sensitive to the choice of covariates.

Numerical verification showed that: (1) χ^2^, CFI, TLI, RMSEA, and SRMR for the five-factor model matched the analysis output; (2) the maximum difference between the five scale means recalculated from the raw items and the Excel scoring columns was 0.000; (3) an independent linear-regression verification differed from the primary analysis by no more than 1.17 × 10^−15^ in β; (4) after changing the bootstrap random seed, the inclusion or exclusion of 0 by the intervals was identical for all 10 direct, total, and specific indirect associations, and the maximum difference between interval endpoints was 0.00164; and (5) after excluding the record flagged for data quality, the full serial indirect association remained 0.036.

## 5 Discussion

### 5.1 Conceptual distinctness and theoretical framing

This study examined the multilevel statistical associations among teaching environments, relational experiences, learning participation, and subjective wellbeing. Based on 815 complete survey records, the sample was randomly divided into an EFA group (*n* = 408) and a CFA group (*n* = 407). Findings from the two groups showed good consistency in the number of factors, item assignments, and measurement structure, providing a relatively stable measurement foundation.

The measurement outcomes support the structural differentiation of these five interlinked constructs. Theoretically, while this study drew upon multiple perspectives, Self-Determination Theory (SDT) served as the primary explanatory mechanism tying these constructs together. Within this framework, a supportive teaching environment represents the external contextual conditions necessary for fulfilling students' basic psychological needs for autonomy, competence, and relatedness. Psychological safety and university belonging function as specific manifestations of fulfilled relatedness, learning engagement emerges as autonomous participation (competence and autonomy), and psychological wellbeing reflects the ultimate state of human flourishing in higher education. Exploring how these specific human-centric pathways sustain student thriving is highly relevant today, as current academic environments are increasingly hybridized and transformed by the rapid integration of artificial intelligence and digital teaching infrastructures, where establishing systemic educational psychological safety is increasingly recognized as a foundational prerequisite for activating students' intrinsic learning engagement (Hu et al., 2026).

### 5.2 Overall association between the teaching environment and psychological wellbeing

The total association between EES and PWB was 0.435, *p* < 0.001; after PS, SB, and LE were included, a direct association of 0.221 remained. Thus, in the survey data, students' favorable evaluations of instructional resources, teacher support, the communication climate, institutional fairness, and feedback channels co-occurred with higher psychological wellbeing scores.

This finding is consistent with the ecological systems and self-determination theory frameworks adopted in the present study. The teaching environment is not a single material condition but an educational context jointly constituted by curricular resources, teacher–student interactions, peer relationships, and institutional norms. A more supportive teaching environment may be concurrently associated with students‘ experiences of autonomy, competence, relatedness, and environmental mastery. This pattern aligns with robust prior evidence showing that broad psychosocial school environments and academic support systems are closely tied to students' socio-emotional development and mental health (Aldridge and McChesney, 2018; Wang and Degol, 2016). Building upon these foundational perspectives, our findings further resonate with recent literature underscoring the critical necessity of continually reassessing psychological wellbeing in evolving higher educational settings, reflecting its profound interconnectedness with holistic student functioning and academic thriving (Mukhlis et al., 2024). The survey finding is directionally consistent with these studies, but it cannot establish that psychological wellbeing changes as a consequence of the teaching environment; rather, it maps a robust covariation within the current sample.

### 5.3 Teaching environment and psychological safety

The standardized conditional association between EES and PS was 0.503, with *f* ^2^ = 0.339, making it one of the stronger focal paths. In the survey data, students' evaluations of the teaching environment were therefore closely associated with whether they felt able to express themselves, ask questions, acknowledge difficulties, and seek help in classroom and campus interactions.

Psychological safety concerns whether individuals can take moderate interpersonal risks without excessive fear of humiliation, rejection, or negative evaluation. How teachers respond to mistakes, whether classrooms permit divergent views, whether evaluation rules are transparent, and whether peers treat one another with respect may all be associated with students' judgments of safety in the teaching environment. Students who perceive greater respect, fairness, and inclusion also generally report higher psychological safety; this concurrent association requires examination using authentic longitudinal data.

Recent evidence confirms that constructive feedback and reflective assessments directly build psychological safety in higher education (McGuire, 2026), and similar dynamics are observed in university project-based learning teams where inclusive peer mechanisms alleviate anxiety (Clausen et al., 2025). These findings resonate with the direction observed for H2 in the present study. The statistical relationship between the teaching environment and psychological wellbeing therefore concerns not only the availability of resources but also how students perceive institutional and relational features of everyday interactions. Psychological safety can be regarded as a relational variable situated adjacently to both constructs in the model.

### 5.4 Psychological safety, university belonging, and learning engagement

The path analysis yielded a standardized conditional association of 0.397 between PS and SB, with *f* ^2^ = 0.165, and a standardized conditional association of 0.455 between SB and LE, with *f* ^2^ = 0.225. Both findings are compatible with the theoretical ordering of “safe participation–membership and belonging–learning engagement,” but do not establish that this sequence actually unfolds over time.

Psychological safety and university belonging are not the same construct. The former concerns students' judgments of the interpersonal risks accompanying speaking up, asking questions, and making mistakes; the latter concerns whether students feel accepted, respected, and recognized by members of their institution. In actual educational experiences, the two may change concurrently: students who are less concerned about the consequences of speaking up often also report more interactions with teachers and peers and a stronger sense of membership, but cross-sectional data cannot establish the direction of this association.

In their meta-analysis, Allen et al. identified school safety, teacher support, peer support, emotional stability, and academic motivation as important factors closely associated with university belonging (Allen et al., 2018). This finding is directionally consistent with the present study and suggests that the close association between experiences of safety and university belonging warrants further investigation. Contemporary evidence across international cohorts continues to reinforce this dynamic, demonstrating that active social interactions and a perceived sense of academic belonging significantly predict elevated multi-dimensional psychological wellbeing (Zhang et al., 2025).

The positive path between university belonging and learning engagement reflects a concurrent association between relational identification and participation in learning. Students who regard themselves as accepted and valued members of the school community also typically report greater participation in coursework and campus life. Pedler et al. (2022) found that university belonging among university students was positively associated with learning engagement, enjoyment of learning, and intentions to persist in their studies. Building upon this, recent comprehensive analyses emphasize that cultivating a supportive teaching and learning environment serves as the vital foundation for fostering this sense of belonging, which subsequently builds holistic psychological resilience alongside engagement (Wang et al., 2026). Psychological safety and university belonging may therefore be important relational variables for understanding differences in learning participation, but they should not be interpreted as established antecedents.

### 5.5 Learning engagement and psychological wellbeing

The standardized conditional association between LE and PWB was 0.395, with *f* ^2^ = 0.168, indicating that vitality, concentration, and sustained participation in learning activities co-occurred with higher psychological wellbeing scores.

Within the conceptual focus of this study, learning engagement occupies a position between experiences of the environment and psychological wellbeing in the pre specified model. The teaching environment, psychological safety, and university belonging primarily reflect students' experiences of the educational context and relational climate, whereas learning engagement reflects a specific state of participation in learning. A sense of purpose, competence, and progress in learning may be concurrently associated with environmental mastery, purpose in life, and personal growth in Ryff's theory of psychological wellbeing.

Utilizing structural models, contemporary studies have increasingly identified learning engagement as a crucial mediating mechanism; for instance, Hamidkholgh et al. (2026) systematically confirmed the mediating role of learning engagement in the relationship between students' emotional management capacities and their subjective wellbeing in higher education. These findings resemble those for the overall model in the present study. Additionally, recent structural equation modeling within complex higher education contexts corroborates that multi-level institutional and social supports fundamentally operate through sustained academic engagement to fortify overarching student psychological wellbeing (Chaudhry et al., 2024).

The direction of the relationship between learning engagement and psychological wellbeing may be reciprocal or influenced by common factors. Lizarte Simón et al. (2024) verified that elevated psychological wellbeing significantly drives sustained academic engagement during university education. Students in better psychological states may report greater energy for learning, while positive learning experiences may co-occur with feelings of competence, purpose, and positive affect. Accordingly, LE → PWB is specified only as the theoretical ordering of the equations used to test the proposed model and is not interpreted as an established unidirectional causal relationship.

### 5.6 Serial association and competing paths

The product term for the full sequence EES → PS → SB → LE → PWB was 0.036, 95% CI [0.026, 0.047], accounting for approximately 8.26% of the total association between EES and PWB. Its bootstrap interval excluded 0, and thus confirmed the presence of this full serial association within the dataset.

The value of the full serial pathway was noticeably smaller than those of some shorter indirect pathways or the direct effect. This small effect magnitude is an expected statistical consequence of multiplying multiple standardized coefficients in a lengthy chain; theoretically, it indicates that in complex educational settings, student wellbeing is not dominated by any single elaborate pathway, but rather is sustained cumulatively through multiple shorter, simultaneous mechanisms. After PS, SB, and LE were included, a direct association of 0.221 remained between EES and PWB, and the total indirect association accounted for approximately 49.15% of the total association, indicating the coexistence of a direct association and multiple indirect associations.

The competing-path analysis further showed that the statistical significance of a cross-sectional product term cannot independently verify the psychological ordering of intermediate variables, as alternative variable arrangements also yielded intervals excluding zero.

Accordingly, the pre specified sequence should not be understood as identifying the only possible order, but as specifying one serial association that is compatible with the survey data under the theoretical framework of the present study. The teaching environment retains a direct relationship with psychological wellbeing and also forms indirect associations of varying lengths with experiences of safety, experiences of belonging, and learning engagement, producing a statistical structure in which multiple pathways coexist.

### 5.7 Theoretical significance, practical implications, and research boundaries

This study integrates ecological systems theory, self-determination theory, psychological safety theory, belongingness theory, learning engagement theory, and Ryff's theory of psychological wellbeing within a single analytical framework. Compared with examining only the bivariate relationship between the teaching environment and psychological wellbeing, this framework further distinguishes perceptions of the external environment, relational psychological experiences, states of learning participation, and positive psychological functioning, thereby providing a multivariable structure for testing in future research using authentic samples.

Practical implications should be drawn cautiously. Because cross-sectional associations cannot establish that changing the teaching environment will inevitably cause improvements in psychological wellbeing, the identified institutional and relational factors should be viewed as potentially modifiable targets for future evidence-based interventions, rather than as strategies with already proven causal effectiveness.

To support student thriving, universities might consider concrete initiatives such as enhancing structured peer learning, developing faculty mentoring systems, actively fostering a sense of community, and building a tangibly supportive campus ecosystem (Gao and Mohamad, 2025). These practices provide a foundation for future inquiries aiming to evaluate true psychological improvements.

It is important to note that the present study used cross-sectional survey data and specified path directions according to a theoretical ordering. The findings describe only the correlational structure among the variables and the fit of the models; they cannot establish temporal ordering, effects in the target population, or causal direction. In addition to the inherent limitations of concurrent measurement, the possibility of reverse directionality exists—students with structurally higher wellbeing, for instance, may evaluate their academic environment more positively. Furthermore, individual confounders such as personality traits, socioeconomic conditions, prior educational achievement, and pre-existing mental health status were not fully accounted for and might influence these relationships. Future studies should use authentic samples and employ longitudinal follow-up, cross-lagged models, or educational intervention designs to further examine the stability, causality, and directionality of the relationships among the five variables.

## 6 Limitations and future directions

This study used a cross-sectional analysis based on a nationwide survey of higher education institutions. Because the data were affected by regional differences, instructional arrangements, examination periods, internship schedules, and other contextual factors, they were subject to the inherent statistical limitations of cross-sectional data and cannot provide definitive proof of temporal or causal directionality. Although participation was voluntary and questionnaires were distributed randomly, sample coverage still varied to some extent.

More importantly, several alternative explanations remain. The possibility of reverse directionality must be acknowledged, as students with higher baseline wellbeing might naturally perceive their academic environments more favorably. Furthermore, unmeasured individual confounders—such as personality traits, socio-economic status, and prior mental health conditions—could synchronously influence both environmental perceptions and subjective wellbeing. Future research will therefore seek to adopt longitudinal or cross-lagged panel methods, continuously track the issues examined here, obtain support from a larger number of cases across diverse demographics, and ultimately utilize educational intervention designs to strengthen the causal robustness of the evidence.

## 7 Conclusion

To elucidate the relationships among the teaching environment, psychological safety, university belonging, learning engagement, and psychological wellbeing, this study surveyed 815 higher education students and comprehensively tested a multilevel serial structural model.

The results showed that: (1) the overall positive association between the teaching environment and psychological wellbeing was substantial; (2) after the three psychological and behavioral variables were included, a significant direct association remained between them; and (3) the full serial indirect association was fully supported (interval excluding zero), and the confidence intervals for all seven specific indirect associations excluded 0. These findings suggest that the teaching environment is directly associated with psychological wellbeing and may also be indirectly related to it through multiple concurrent routes.

As an external educational condition, the teaching environment constitutes an important psychological context for students' sense of safety, sense of belonging, and learning engagement. The association between the teaching environment and psychological safety was relatively strong. The findings suggest a continuous psychological associative structure of “environmental perceptions–psychological safety–relational belonging–behavioral engagement–psychological wellbeing,” indicating a deep connection between external environmental resources and students' internal psychological functioning.

Thus, students' psychological wellbeing should be understood not only from the perspective of individual affect but also in relation to the teaching environment, university belonging, psychological safety, and learning engagement. Higher education institutions should regularly assess students' mental health, foster a supportive teaching environment, improve teacher–student relationships, and promote students' sense of belonging and participation in learning. Because the study used cross-sectional data, the results reflect an associative structure among the variables and do not represent definitive causal relationships. Nevertheless, they provide valuable empirical reference points for educational psychological assessment, offering potentially modifiable targets for tiered psychological support within future higher education teaching practice.

## Data Availability

The original contributions presented in the study are included in the article/Supplementary material, further inquiries can be directed to the corresponding author.
