# Supplementary material for: The predictive association between the teaching environment and psychological wellbeing: psychological safety, university belonging, and learning engagement
Source: Front Psychol. 2026 Oct 20;17:1983869. doi: 10.3389/fpsyg.2026.1983869 (PMC13727545; doi:10.3389/fpsyg.2026.1983869)
Supplement: Supplementary file 1 [file Data_Sheet_1.zip › Supplementary Material 2_Cross Tabulations and Chi-Square Tests.docx]

**Cross-Tabulations and Chi-Square Tests of Background Variables**

Teaching Environment and Psychological Well-Being Study · N = 815

# 1. Test Description

Based on 815 data records, this document separately reports 28 pairwise cross-tabulations among eight background variables: gender, age, level of study, academic field, living arrangement, academic performance, academic stress, and frequency of psychological distress. To reduce the influence of sparse cells, the inferential analyses combined the categories 24–26 years and 27 years or older as '24 years or older' and combined master's and doctoral students as 'graduate students'; the original categories of all other variables were retained. Cross-tabulation cells show n (column %), together with Pearson's χ², degrees of freedom, the two-sided p value, Cramér's V, the minimum expected frequency, and the proportion of cells with E < 5.

# 2. Overview of Results

After sparse categories for age and level of study were combined, the eight background variables yielded 28 pairwise cross-tabulations. Six associations reached the unadjusted 0.05 significance level: gender × age, gender × academic stress, gender × frequency of psychological distress, age × level of study, academic performance × frequency of psychological distress, and academic stress × frequency of psychological distress. The association between age and level of study was strongest (V = 0.446), followed by the association between academic stress and frequency of psychological distress (V = 0.289); V ranged from 0.099 to 0.123 for the remaining significant associations. The minimum expected frequency across the 28 cross-tabulations was 5.466, no cells had E < 5, and Pearson's chi-square test was used throughout, as shown in Table A.

Table A. Cross-Tabulation Tests of Control Variables

| **Variable Pair** | **χ²** | **df** | **p** | **V** | **Minimum E** | **E<5** | **Test** |
| --- | --- | --- | --- | --- | --- | --- | --- |
| Gender × Age | 7.968 | 2 | 0.019 | 0.099 | 48.785 | 0/6 | Pearson |
| Gender × Level of Study | 2.839 | 2 | 0.242 | 0.059 | 35.282 | 0/6 | Pearson |
| Gender × Academic Field | 3.648 | 3 | 0.302 | 0.067 | 38.331 | 0/8 | Pearson |
| Gender × Living Arrangement | 3.493 | 3 | 0.322 | 0.065 | 23.957 | 0/8 | Pearson |
| Gender × Academic Performance | 3.594 | 3 | 0.309 | 0.066 | 69.693 | 0/8 | Pearson |
| Gender × Academic Stress | 10.234 | 3 | 0.017 | 0.112 | 64.466 | 0/8 | Pearson |
| Gender × Frequency of Psychological Distress | 12.336 | 3 | 0.006 | 0.123 | 56.190 | 0/8 | Pearson |
| Age × Level of Study | 323.706 | 4 | <0.001 | 0.446 | 11.131 | 0/9 | Pearson |
| Age × Academic Field | 7.061 | 6 | 0.315 | 0.066 | 12.093 | 0/12 | Pearson |
| Age × Living Arrangement | 2.944 | 6 | 0.816 | 0.042 | 7.558 | 0/12 | Pearson |
| Age × Academic Performance | 6.789 | 6 | 0.341 | 0.065 | 21.988 | 0/12 | Pearson |
| Age × Academic Stress | 2.295 | 6 | 0.891 | 0.038 | 20.339 | 0/12 | Pearson |
| Age × Frequency of Psychological Distress | 3.468 | 6 | 0.748 | 0.046 | 17.728 | 0/12 | Pearson |
| Level of Study × Academic Field | 1.142 | 6 | 0.980 | 0.026 | 8.746 | 0/12 | Pearson |
| Level of Study × Living Arrangement | 3.692 | 6 | 0.718 | 0.048 | 5.466 | 0/12 | Pearson |
| Level of Study × Academic Performance | 5.970 | 6 | 0.427 | 0.061 | 15.902 | 0/12 | Pearson |
| Level of Study × Academic Stress | 11.749 | 6 | 0.068 | 0.085 | 14.709 | 0/12 | Pearson |
| Level of Study × Frequency of Psychological Distress | 4.072 | 6 | 0.667 | 0.050 | 12.821 | 0/12 | Pearson |
| Academic Field × Living Arrangement | 6.221 | 9 | 0.718 | 0.050 | 5.939 | 0/16 | Pearson |
| Academic Field × Academic Performance | 9.571 | 9 | 0.386 | 0.063 | 17.276 | 0/16 | Pearson |
| Academic Field × Academic Stress | 9.522 | 9 | 0.391 | 0.062 | 15.980 | 0/16 | Pearson |
| Academic Field × Frequency of Psychological Distress | 9.865 | 9 | 0.362 | 0.064 | 13.929 | 0/16 | Pearson |
| Living Arrangement × Academic Performance | 12.572 | 9 | 0.183 | 0.072 | 10.798 | 0/16 | Pearson |
| Living Arrangement × Academic Stress | 12.190 | 9 | 0.203 | 0.071 | 9.988 | 0/16 | Pearson |
| Living Arrangement × Frequency of Psychological Distress | 10.404 | 9 | 0.319 | 0.065 | 8.706 | 0/16 | Pearson |
| Academic Performance × Academic Stress | 12.908 | 9 | 0.167 | 0.073 | 29.055 | 0/16 | Pearson |
| Academic Performance × Frequency of Psychological Distress | 27.839 | 9 | 0.001 | 0.107 | 25.325 | 0/16 | Pearson |
| Academic Stress × Frequency of Psychological Distress | 204.099 | 9 | <0.001 | 0.289 | 23.426 | 0/16 | Pearson |

Note. Age and level of study use the merged three-category classifications; all other variables retain their original categories. p is the unadjusted two-sided asymptotic value from Pearson's chi-square test; V is Cramér's V; and E < 5 is the number of cells with an expected frequency below 5 divided by the total number of cells. All minimum expected frequencies were ≥ 5.466; therefore, no exact tests were required. The complete cross-tabulations are presented in Section 3 (Tables 1–28).

# 3. Complete Cross-Tabulations

The 28 cross-tabulations below follow the variable-coding order used in Appendix B of the manuscript. Age was grouped as 18–20 years, 21–23 years, and 24 years or older, while level of study was grouped as junior college, undergraduate, and graduate; all other variables retained their original categories. For verification purposes, each table includes the test statistic, effect size, and expected-frequency diagnostics.

Table 1. Gender × Age

| **Variable** | **Category** | **18–20 years** | **21–23 years** | **24 years or older** | **Total** | **χ²(df)** | **p** |
| --- | --- | --- | --- | --- | --- | --- | --- |
| Gender | Male | 200(46.95) | 119(42.96) | 36(32.14) | 355(43.56) | 7.968(2) | 0.019 |
|  | Female | 226(53.05) | 158(57.04) | 76(67.86) | 460(56.44) | 7.968(2) | 0.019 |
|  | Total | 426 | 277 | 112 | 815 |  |  |

Note. Cells show n (column %). Cramér's V = 0.099; minimum expected frequency = 48.785; cells with expected frequencies < 5 = 0/6. p is the two-sided asymptotic value from Pearson's chi-square test.

Table 2. Gender × Level of Study

| **Variable** | **Category** | **Junior college** | **Undergraduate** | **Graduate** | **Total** | **χ²(df)** | **p** |
| --- | --- | --- | --- | --- | --- | --- | --- |
| Gender | Male | 40(49.38) | 262(44.03) | 53(38.13) | 355(43.56) | 2.839(2) | 0.242 |
|  | Female | 41(50.62) | 333(55.97) | 86(61.87) | 460(56.44) | 2.839(2) | 0.242 |
|  | Total | 81 | 595 | 139 | 815 |  |  |

Note. Cells show n (column %). Cramér's V = 0.059; minimum expected frequency = 35.282; cells with expected frequencies < 5 = 0/6. p is the two-sided asymptotic value from Pearson's chi-square test.

Table 3. Gender × Academic Field

| **Variable** | **Category** | **Humanities and Social Sciences** | **Science and Engineering** | **Medicine or Agriculture** | **Arts or Sports** | **Total** | **χ²(df)** | **p** |
| --- | --- | --- | --- | --- | --- | --- | --- | --- |
| Gender | Male | 137(44.77) | 123(45.05) | 65(43.92) | 30(34.09) | 355(43.56) | 3.648(3) | 0.302 |
|  | Female | 169(55.23) | 150(54.95) | 83(56.08) | 58(65.91) | 460(56.44) | 3.648(3) | 0.302 |
|  | Total | 306 | 273 | 148 | 88 | 815 |  |  |

Note. Cells show n (column %). Cramér's V = 0.067; minimum expected frequency = 38.331; cells with expected frequencies < 5 = 0/8. p is the two-sided asymptotic value from Pearson's chi-square test.

Table 4. Gender × Living Arrangement

| **Variable** | **Category** | **On-campus residence** | **Off-campus rental accommodation** | **Living with family** | **Other** | **Total** | **χ²(df)** | **p** |
| --- | --- | --- | --- | --- | --- | --- | --- | --- |
| Gender | Male | 170(45.70) | 72(37.89) | 87(43.94) | 26(47.27) | 355(43.56) | 3.493(3) | 0.322 |
|  | Female | 202(54.30) | 118(62.11) | 111(56.06) | 29(52.73) | 460(56.44) | 3.493(3) | 0.322 |
|  | Total | 372 | 190 | 198 | 55 | 815 |  |  |

Note. Cells show n (column %). Cramér's V = 0.065; minimum expected frequency = 23.957; cells with expected frequencies < 5 = 0/8. p is the two-sided asymptotic value from Pearson's chi-square test.

Table 5. Gender × Academic Performance

| **Variable** | **Category** | **Poor** | **Average** | **Good** | **Excellent** | **Total** | **χ²(df)** | **p** |
| --- | --- | --- | --- | --- | --- | --- | --- | --- |
| Gender | Male | 94(49.47) | 98(42.42) | 97(41.45) | 66(41.25) | 355(43.56) | 3.594(3) | 0.309 |
|  | Female | 96(50.53) | 133(57.58) | 137(58.55) | 94(58.75) | 460(56.44) | 3.594(3) | 0.309 |
|  | Total | 190 | 231 | 234 | 160 | 815 |  |  |

Note. Cells show n (column %). Cramér's V = 0.066; minimum expected frequency = 69.693; cells with expected frequencies < 5 = 0/8. p is the two-sided asymptotic value from Pearson's chi-square test.

Table 6. Gender × Academic Stress

| **Variable** | **Category** | **Very low** | **Low** | **High** | **Very high** | **Total** | **χ²(df)** | **p** |
| --- | --- | --- | --- | --- | --- | --- | --- | --- |
| Gender | Male | 71(41.28) | 95(36.82) | 119(50.21) | 70(47.30) | 355(43.56) | 10.234(3) | 0.017 |
|  | Female | 101(58.72) | 163(63.18) | 118(49.79) | 78(52.70) | 460(56.44) | 10.234(3) | 0.017 |
|  | Total | 172 | 258 | 237 | 148 | 815 |  |  |

Note. Cells show n (column %). Cramér's V = 0.112; minimum expected frequency = 64.466; cells with expected frequencies < 5 = 0/8. p is the two-sided asymptotic value from Pearson's chi-square test.

Table 7. Gender × Frequency of Psychological Distress

| **Variable** | **Category** | **Never** | **Occasionally** | **Often** | **Almost always** | **Total** | **χ²(df)** | **p** |
| --- | --- | --- | --- | --- | --- | --- | --- | --- |
| Gender | Male | 72(41.86) | 110(39.43) | 99(42.13) | 74(57.36) | 355(43.56) | 12.336(3) | 0.006 |
|  | Female | 100(58.14) | 169(60.57) | 136(57.87) | 55(42.64) | 460(56.44) | 12.336(3) | 0.006 |
|  | Total | 172 | 279 | 235 | 129 | 815 |  |  |

Note. Cells show n (column %). Cramér's V = 0.123; minimum expected frequency = 56.190; cells with expected frequencies < 5 = 0/8. p is the two-sided asymptotic value from Pearson's chi-square test.

Table 8. Age × Level of Study

| **Variable** | **Category** | **Junior college** | **Undergraduate** | **Graduate** | **Total** | **χ²(df)** | **p** |
| --- | --- | --- | --- | --- | --- | --- | --- |
| Age | 18–20 years | 66(81.48) | 351(58.99) | 9(6.47) | 426(52.27) | 323.706(4) | <0.001 |
|  | 21–23 years | 15(18.52) | 213(35.80) | 49(35.25) | 277(33.99) | 323.706(4) | <0.001 |
|  | 24 years or older | 0(0.00) | 31(5.21) | 81(58.27) | 112(13.74) | 323.706(4) | <0.001 |
|  | Total | 81 | 595 | 139 | 815 |  |  |

Note. Cells show n (column %). Cramér's V = 0.446; minimum expected frequency = 11.131; cells with expected frequencies < 5 = 0/9. p is the two-sided asymptotic value from Pearson's chi-square test.

Table 9. Age × Academic Field

| **Variable** | **Category** | **Humanities and Social Sciences** | **Science and Engineering** | **Medicine or Agriculture** | **Arts or Sports** | **Total** | **χ²(df)** | **p** |
| --- | --- | --- | --- | --- | --- | --- | --- | --- |
| Age | 18–20 years | 173(56.54) | 142(52.01) | 69(46.62) | 42(47.73) | 426(52.27) | 7.061(6) | 0.315 |
|  | 21–23 years | 92(30.07) | 90(32.97) | 60(40.54) | 35(39.77) | 277(33.99) | 7.061(6) | 0.315 |
|  | 24 years or older | 41(13.40) | 41(15.02) | 19(12.84) | 11(12.50) | 112(13.74) | 7.061(6) | 0.315 |
|  | Total | 306 | 273 | 148 | 88 | 815 |  |  |

Note. Cells show n (column %). Cramér's V = 0.066; minimum expected frequency = 12.093; cells with expected frequencies < 5 = 0/12. p is the two-sided asymptotic value from Pearson's chi-square test.

Table 10. Age × Living Arrangement

| **Variable** | **Category** | **On-campus residence** | **Off-campus rental accommodation** | **Living with family** | **Other** | **Total** | **χ²(df)** | **p** |
| --- | --- | --- | --- | --- | --- | --- | --- | --- |
| Age | 18–20 years | 187(50.27) | 108(56.84) | 103(52.02) | 28(50.91) | 426(52.27) | 2.944(6) | 0.816 |
|  | 21–23 years | 130(34.95) | 59(31.05) | 67(33.84) | 21(38.18) | 277(33.99) | 2.944(6) | 0.816 |
|  | 24 years or older | 55(14.78) | 23(12.11) | 28(14.14) | 6(10.91) | 112(13.74) | 2.944(6) | 0.816 |
|  | Total | 372 | 190 | 198 | 55 | 815 |  |  |

Note. Cells show n (column %). Cramér's V = 0.042; minimum expected frequency = 7.558; cells with expected frequencies < 5 = 0/12. p is the two-sided asymptotic value from Pearson's chi-square test.

Table 11. Age × Academic Performance

| **Variable** | **Category** | **Poor** | **Average** | **Good** | **Excellent** | **Total** | **χ²(df)** | **p** |
| --- | --- | --- | --- | --- | --- | --- | --- | --- |
| Age | 18–20 years | 111(58.42) | 123(53.25) | 117(50.00) | 75(46.88) | 426(52.27) | 6.789(6) | 0.341 |
|  | 21–23 years | 56(29.47) | 73(31.60) | 84(35.90) | 64(40.00) | 277(33.99) | 6.789(6) | 0.341 |
|  | 24 years or older | 23(12.11) | 35(15.15) | 33(14.10) | 21(13.12) | 112(13.74) | 6.789(6) | 0.341 |
|  | Total | 190 | 231 | 234 | 160 | 815 |  |  |

Note. Cells show n (column %). Cramér's V = 0.065; minimum expected frequency = 21.988; cells with expected frequencies < 5 = 0/12. p is the two-sided asymptotic value from Pearson's chi-square test.

Table 12. Age × Academic Stress

| **Variable** | **Category** | **Very low** | **Low** | **High** | **Very high** | **Total** | **χ²(df)** | **p** |
| --- | --- | --- | --- | --- | --- | --- | --- | --- |
| Age | 18–20 years | 92(53.49) | 130(50.39) | 124(52.32) | 80(54.05) | 426(52.27) | 2.295(6) | 0.891 |
|  | 21–23 years | 55(31.98) | 94(36.43) | 83(35.02) | 45(30.41) | 277(33.99) | 2.295(6) | 0.891 |
|  | 24 years or older | 25(14.53) | 34(13.18) | 30(12.66) | 23(15.54) | 112(13.74) | 2.295(6) | 0.891 |
|  | Total | 172 | 258 | 237 | 148 | 815 |  |  |

Note. Cells show n (column %). Cramér's V = 0.038; minimum expected frequency = 20.339; cells with expected frequencies < 5 = 0/12. p is the two-sided asymptotic value from Pearson's chi-square test.

Table 13. Age × Frequency of Psychological Distress

| **Variable** | **Category** | **Never** | **Occasionally** | **Often** | **Almost always** | **Total** | **χ²(df)** | **p** |
| --- | --- | --- | --- | --- | --- | --- | --- | --- |
| Age | 18–20 years | 89(51.74) | 145(51.97) | 122(51.91) | 70(54.26) | 426(52.27) | 3.468(6) | 0.748 |
|  | 21–23 years | 56(32.56) | 91(32.62) | 87(37.02) | 43(33.33) | 277(33.99) | 3.468(6) | 0.748 |
|  | 24 years or older | 27(15.70) | 43(15.41) | 26(11.06) | 16(12.40) | 112(13.74) | 3.468(6) | 0.748 |
|  | Total | 172 | 279 | 235 | 129 | 815 |  |  |

Note. Cells show n (column %). Cramér's V = 0.046; minimum expected frequency = 17.728; cells with expected frequencies < 5 = 0/12. p is the two-sided asymptotic value from Pearson's chi-square test.

Table 14. Level of Study × Academic Field

| **Variable** | **Category** | **Humanities and Social Sciences** | **Science and Engineering** | **Medicine or Agriculture** | **Arts or Sports** | **Total** | **χ²(df)** | **p** |
| --- | --- | --- | --- | --- | --- | --- | --- | --- |
| Level of Study | Junior college | 33(10.78) | 25(9.16) | 16(10.81) | 7(7.95) | 81(9.94) | 1.142(6) | 0.980 |
|  | Undergraduate | 221(72.22) | 201(73.63) | 106(71.62) | 67(76.14) | 595(73.01) | 1.142(6) | 0.980 |
|  | Graduate | 52(16.99) | 47(17.22) | 26(17.57) | 14(15.91) | 139(17.06) | 1.142(6) | 0.980 |
|  | Total | 306 | 273 | 148 | 88 | 815 |  |  |

Note. Cells show n (column %). Cramér's V = 0.026; minimum expected frequency = 8.746; cells with expected frequencies < 5 = 0/12. p is the two-sided asymptotic value from Pearson's chi-square test.

Table 15. Level of Study × Living Arrangement

| **Variable** | **Category** | **On-campus residence** | **Off-campus rental accommodation** | **Living with family** | **Other** | **Total** | **χ²(df)** | **p** |
| --- | --- | --- | --- | --- | --- | --- | --- | --- |
| Level of Study | Junior college | 33(8.87) | 23(12.11) | 18(9.09) | 7(12.73) | 81(9.94) | 3.692(6) | 0.718 |
|  | Undergraduate | 276(74.19) | 137(72.11) | 141(71.21) | 41(74.55) | 595(73.01) | 3.692(6) | 0.718 |
|  | Graduate | 63(16.94) | 30(15.79) | 39(19.70) | 7(12.73) | 139(17.06) | 3.692(6) | 0.718 |
|  | Total | 372 | 190 | 198 | 55 | 815 |  |  |

Note. Cells show n (column %). Cramér's V = 0.048; minimum expected frequency = 5.466; cells with expected frequencies < 5 = 0/12. p is the two-sided asymptotic value from Pearson's chi-square test.

Table 16. Level of Study × Academic Performance

| **Variable** | **Category** | **Poor** | **Average** | **Good** | **Excellent** | **Total** | **χ²(df)** | **p** |
| --- | --- | --- | --- | --- | --- | --- | --- | --- |
| Level of Study | Junior college | 19(10.00) | 25(10.82) | 21(8.97) | 16(10.00) | 81(9.94) | 5.970(6) | 0.427 |
|  | Undergraduate | 147(77.37) | 158(68.40) | 171(73.08) | 119(74.38) | 595(73.01) | 5.970(6) | 0.427 |
|  | Graduate | 24(12.63) | 48(20.78) | 42(17.95) | 25(15.62) | 139(17.06) | 5.970(6) | 0.427 |
|  | Total | 190 | 231 | 234 | 160 | 815 |  |  |

Note. Cells show n (column %). Cramér's V = 0.061; minimum expected frequency = 15.902; cells with expected frequencies < 5 = 0/12. p is the two-sided asymptotic value from Pearson's chi-square test.

Table 17. Level of Study × Academic Stress

| **Variable** | **Category** | **Very low** | **Low** | **High** | **Very high** | **Total** | **χ²(df)** | **p** |
| --- | --- | --- | --- | --- | --- | --- | --- | --- |
| Level of Study | Junior college | 9(5.23) | 26(10.08) | 30(12.66) | 16(10.81) | 81(9.94) | 11.749(6) | 0.068 |
|  | Undergraduate | 140(81.40) | 191(74.03) | 159(67.09) | 105(70.95) | 595(73.01) | 11.749(6) | 0.068 |
|  | Graduate | 23(13.37) | 41(15.89) | 48(20.25) | 27(18.24) | 139(17.06) | 11.749(6) | 0.068 |
|  | Total | 172 | 258 | 237 | 148 | 815 |  |  |

Note. Cells show n (column %). Cramér's V = 0.085; minimum expected frequency = 14.709; cells with expected frequencies < 5 = 0/12. p is the two-sided asymptotic value from Pearson's chi-square test.

Table 18. Level of Study × Frequency of Psychological Distress

| **Variable** | **Category** | **Never** | **Occasionally** | **Often** | **Almost always** | **Total** | **χ²(df)** | **p** |
| --- | --- | --- | --- | --- | --- | --- | --- | --- |
| Level of Study | Junior college | 12(6.98) | 27(9.68) | 25(10.64) | 17(13.18) | 81(9.94) | 4.072(6) | 0.667 |
|  | Undergraduate | 131(76.16) | 200(71.68) | 172(73.19) | 92(71.32) | 595(73.01) | 4.072(6) | 0.667 |
|  | Graduate | 29(16.86) | 52(18.64) | 38(16.17) | 20(15.50) | 139(17.06) | 4.072(6) | 0.667 |
|  | Total | 172 | 279 | 235 | 129 | 815 |  |  |

Note. Cells show n (column %). Cramér's V = 0.050; minimum expected frequency = 12.821; cells with expected frequencies < 5 = 0/12. p is the two-sided asymptotic value from Pearson's chi-square test.

Table 19. Academic Field × Living Arrangement

| **Variable** | **Category** | **On-campus residence** | **Off-campus rental accommodation** | **Living with family** | **Other** | **Total** | **χ²(df)** | **p** |
| --- | --- | --- | --- | --- | --- | --- | --- | --- |
| Academic Field | Humanities and Social Sciences | 134(36.02) | 71(37.37) | 85(42.93) | 16(29.09) | 306(37.55) | 6.221(9) | 0.718 |
|  | Science and Engineering | 129(34.68) | 60(31.58) | 64(32.32) | 20(36.36) | 273(33.50) | 6.221(9) | 0.718 |
|  | Medicine or Agriculture | 71(19.09) | 37(19.47) | 29(14.65) | 11(20.00) | 148(18.16) | 6.221(9) | 0.718 |
|  | Arts or Sports | 38(10.22) | 22(11.58) | 20(10.10) | 8(14.55) | 88(10.80) | 6.221(9) | 0.718 |
|  | Total | 372 | 190 | 198 | 55 | 815 |  |  |

Note. Cells show n (column %). Cramér's V = 0.050; minimum expected frequency = 5.939; cells with expected frequencies < 5 = 0/16. p is the two-sided asymptotic value from Pearson's chi-square test.

Table 20. Academic Field × Academic Performance

| **Variable** | **Category** | **Poor** | **Average** | **Good** | **Excellent** | **Total** | **χ²(df)** | **p** |
| --- | --- | --- | --- | --- | --- | --- | --- | --- |
| Academic Field | Humanities and Social Sciences | 71(37.37) | 87(37.66) | 90(38.46) | 58(36.25) | 306(37.55) | 9.571(9) | 0.386 |
|  | Science and Engineering | 68(35.79) | 65(28.14) | 88(37.61) | 52(32.50) | 273(33.50) | 9.571(9) | 0.386 |
|  | Medicine or Agriculture | 34(17.89) | 47(20.35) | 37(15.81) | 30(18.75) | 148(18.16) | 9.571(9) | 0.386 |
|  | Arts or Sports | 17(8.95) | 32(13.85) | 19(8.12) | 20(12.50) | 88(10.80) | 9.571(9) | 0.386 |
|  | Total | 190 | 231 | 234 | 160 | 815 |  |  |

Note. Cells show n (column %). Cramér's V = 0.063; minimum expected frequency = 17.276; cells with expected frequencies < 5 = 0/16. p is the two-sided asymptotic value from Pearson's chi-square test.

Table 21. Academic Field × Academic Stress

| **Variable** | **Category** | **Very low** | **Low** | **High** | **Very high** | **Total** | **χ²(df)** | **p** |
| --- | --- | --- | --- | --- | --- | --- | --- | --- |
| Academic Field | Humanities and Social Sciences | 63(36.63) | 99(38.37) | 93(39.24) | 51(34.46) | 306(37.55) | 9.522(9) | 0.391 |
|  | Science and Engineering | 60(34.88) | 92(35.66) | 77(32.49) | 44(29.73) | 273(33.50) | 9.522(9) | 0.391 |
|  | Medicine or Agriculture | 32(18.60) | 36(13.95) | 42(17.72) | 38(25.68) | 148(18.16) | 9.522(9) | 0.391 |
|  | Arts or Sports | 17(9.88) | 31(12.02) | 25(10.55) | 15(10.14) | 88(10.80) | 9.522(9) | 0.391 |
|  | Total | 172 | 258 | 237 | 148 | 815 |  |  |

Note. Cells show n (column %). Cramér's V = 0.062; minimum expected frequency = 15.980; cells with expected frequencies < 5 = 0/16. p is the two-sided asymptotic value from Pearson's chi-square test.

Table 22. Academic Field × Frequency of Psychological Distress

| **Variable** | **Category** | **Never** | **Occasionally** | **Often** | **Almost always** | **Total** | **χ²(df)** | **p** |
| --- | --- | --- | --- | --- | --- | --- | --- | --- |
| Academic Field | Humanities and Social Sciences | 63(36.63) | 105(37.63) | 95(40.43) | 43(33.33) | 306(37.55) | 9.865(9) | 0.362 |
|  | Science and Engineering | 55(31.98) | 96(34.41) | 74(31.49) | 48(37.21) | 273(33.50) | 9.865(9) | 0.362 |
|  | Medicine or Agriculture | 39(22.67) | 40(14.34) | 43(18.30) | 26(20.16) | 148(18.16) | 9.865(9) | 0.362 |
|  | Arts or Sports | 15(8.72) | 38(13.62) | 23(9.79) | 12(9.30) | 88(10.80) | 9.865(9) | 0.362 |
|  | Total | 172 | 279 | 235 | 129 | 815 |  |  |

Note. Cells show n (column %). Cramér's V = 0.064; minimum expected frequency = 13.929; cells with expected frequencies < 5 = 0/16. p is the two-sided asymptotic value from Pearson's chi-square test.

Table 23. Living Arrangement × Academic Performance

| **Variable** | **Category** | **Poor** | **Average** | **Good** | **Excellent** | **Total** | **χ²(df)** | **p** |
| --- | --- | --- | --- | --- | --- | --- | --- | --- |
| Living Arrangement | On-campus residence | 77(40.53) | 112(48.48) | 111(47.44) | 72(45.00) | 372(45.64) | 12.572(9) | 0.183 |
|  | Off-campus rental accommodation | 39(20.53) | 59(25.54) | 56(23.93) | 36(22.50) | 190(23.31) | 12.572(9) | 0.183 |
|  | Living with family | 53(27.89) | 49(21.21) | 53(22.65) | 43(26.88) | 198(24.29) | 12.572(9) | 0.183 |
|  | Other | 21(11.05) | 11(4.76) | 14(5.98) | 9(5.62) | 55(6.75) | 12.572(9) | 0.183 |
|  | Total | 190 | 231 | 234 | 160 | 815 |  |  |

Note. Cells show n (column %). Cramér's V = 0.072; minimum expected frequency = 10.798; cells with expected frequencies < 5 = 0/16. p is the two-sided asymptotic value from Pearson's chi-square test.

Table 24. Living Arrangement × Academic Stress

| **Variable** | **Category** | **Very low** | **Low** | **High** | **Very high** | **Total** | **χ²(df)** | **p** |
| --- | --- | --- | --- | --- | --- | --- | --- | --- |
| Living Arrangement | On-campus residence | 67(38.95) | 123(47.67) | 116(48.95) | 66(44.59) | 372(45.64) | 12.190(9) | 0.203 |
|  | Off-campus rental accommodation | 42(24.42) | 51(19.77) | 62(26.16) | 35(23.65) | 190(23.31) | 12.190(9) | 0.203 |
|  | Living with family | 50(29.07) | 66(25.58) | 49(20.68) | 33(22.30) | 198(24.29) | 12.190(9) | 0.203 |
|  | Other | 13(7.56) | 18(6.98) | 10(4.22) | 14(9.46) | 55(6.75) | 12.190(9) | 0.203 |
|  | Total | 172 | 258 | 237 | 148 | 815 |  |  |

Note. Cells show n (column %). Cramér's V = 0.071; minimum expected frequency = 9.988; cells with expected frequencies < 5 = 0/16. p is the two-sided asymptotic value from Pearson's chi-square test.

Table 25. Living Arrangement × Frequency of Psychological Distress

| **Variable** | **Category** | **Never** | **Occasionally** | **Often** | **Almost always** | **Total** | **χ²(df)** | **p** |
| --- | --- | --- | --- | --- | --- | --- | --- | --- |
| Living Arrangement | On-campus residence | 84(48.84) | 116(41.58) | 114(48.51) | 58(44.96) | 372(45.64) | 10.404(9) | 0.319 |
|  | Off-campus rental accommodation | 34(19.77) | 77(27.60) | 43(18.30) | 36(27.91) | 190(23.31) | 10.404(9) | 0.319 |
|  | Living with family | 44(25.58) | 65(23.30) | 61(25.96) | 28(21.71) | 198(24.29) | 10.404(9) | 0.319 |
|  | Other | 10(5.81) | 21(7.53) | 17(7.23) | 7(5.43) | 55(6.75) | 10.404(9) | 0.319 |
|  | Total | 172 | 279 | 235 | 129 | 815 |  |  |

Note. Cells show n (column %). Cramér's V = 0.065; minimum expected frequency = 8.706; cells with expected frequencies < 5 = 0/16. p is the two-sided asymptotic value from Pearson's chi-square test.

Table 26. Academic Performance × Academic Stress

| **Variable** | **Category** | **Very low** | **Low** | **High** | **Very high** | **Total** | **χ²(df)** | **p** |
| --- | --- | --- | --- | --- | --- | --- | --- | --- |
| Academic Stress | Poor | 42(24.42) | 55(21.32) | 49(20.68) | 44(29.73) | 190(23.31) | 12.908(9) | 0.167 |
|  | Average | 45(26.16) | 73(28.29) | 69(29.11) | 44(29.73) | 231(28.34) | 12.908(9) | 0.167 |
|  | Good | 44(25.58) | 74(28.68) | 81(34.18) | 35(23.65) | 234(28.71) | 12.908(9) | 0.167 |
|  | Excellent | 41(23.84) | 56(21.71) | 38(16.03) | 25(16.89) | 160(19.63) | 12.908(9) | 0.167 |
|  | Total | 172 | 258 | 237 | 148 | 815 |  |  |

Note. Cells show n (column %). Cramér's V = 0.073; minimum expected frequency = 29.055; cells with expected frequencies < 5 = 0/16. p is the two-sided asymptotic value from Pearson's chi-square test.

Table 27. Academic Performance × Frequency of Psychological Distress

| **Variable** | **Category** | **Never** | **Occasionally** | **Often** | **Almost always** | **Total** | **χ²(df)** | **p** |
| --- | --- | --- | --- | --- | --- | --- | --- | --- |
| Academic Stress | Poor | 30(17.44) | 60(21.51) | 58(24.68) | 42(32.56) | 190(23.31) | 27.839(9) | 0.001 |
|  | Average | 55(31.98) | 68(24.37) | 69(29.36) | 39(30.23) | 231(28.34) | 27.839(9) | 0.001 |
|  | Good | 45(26.16) | 80(28.67) | 73(31.06) | 36(27.91) | 234(28.71) | 27.839(9) | 0.001 |
|  | Excellent | 42(24.42) | 71(25.45) | 35(14.89) | 12(9.30) | 160(19.63) | 27.839(9) | 0.001 |
|  | Total | 172 | 279 | 235 | 129 | 815 |  |  |

Note. Cells show n (column %). Cramér's V = 0.107; minimum expected frequency = 25.325; cells with expected frequencies < 5 = 0/16. p is the two-sided asymptotic value from Pearson's chi-square test.

Table 28. Academic Stress × Frequency of Psychological Distress

| **Variable** | **Category** | **Never** | **Occasionally** | **Often** | **Almost always** | **Total** | **χ²(df)** | **p** |
| --- | --- | --- | --- | --- | --- | --- | --- | --- |
| Academic Stress | Very low | 77(44.77) | 67(24.01) | 24(10.21) | 4(3.10) | 172(21.10) | 204.099(9) | <0.001 |
|  | Low | 60(34.88) | 114(40.86) | 63(26.81) | 21(16.28) | 258(31.66) | 204.099(9) | <0.001 |
|  | High | 26(15.12) | 74(26.52) | 90(38.30) | 47(36.43) | 237(29.08) | 204.099(9) | <0.001 |
|  | Very high | 9(5.23) | 24(8.60) | 58(24.68) | 57(44.19) | 148(18.16) | 204.099(9) | <0.001 |
|  | Total | 172 | 279 | 235 | 129 | 815 |  |  |

Note. Cells show n (column %). Cramér's V = 0.289; minimum expected frequency = 23.426; cells with expected frequencies < 5 = 0/16. p is the two-sided asymptotic value from Pearson's chi-square test.
