# Supplementary material for: The predictive association between the teaching environment and psychological wellbeing: psychological safety, university belonging, and learning engagement
Source: Front Psychol. 2026 Oct 20;17:1983869. doi: 10.3389/fpsyg.2026.1983869 (PMC13727545; doi:10.3389/fpsyg.2026.1983869)
Supplement: Supplementary file 1 [file Data_Sheet_1.zip › Supplementary Material 3_Full Survey Instrument.docx]

The Relationship Between the Teaching Environment and Psychological Well-Being:The Serial Indirect Association Through Psychological Safety, University Belonging, and Learning Engagement

| **Target Participants** | Students aged 18 years or older who are currently enrolled at a higher education institution | **Estimated Time** | Approximately 8–10 minutes |
| --- | --- | --- | --- |
| **Reference Period** | The current semester | **Response Format** | Anonymous and voluntary; there are no right or wrong answers |

# Instructions and Informed Consent

Hello. This study aims to understand students' experiences of the teaching environment and how these experiences are associated with psychological well-being. The questionnaire does not collect names, student identification numbers, or contact details. The data will be used solely for academic research and reported only in aggregate form. Participation is entirely voluntary, and you may stop at any time without any adverse consequences. Please answer on the basis of your actual experiences during the current semester. For background questions concerning experiences during the past month or past year, please use the period specified in the question.

**By continuing, you confirm that you have read the information above, voluntarily agree to participate in this study, and consent to the researcher's use of your de-identified questionnaire data. If you have questions about the study, please contact the principal investigator: ____________; email: ____________.**

| **Item** | **Screening Question** | **Response** |
| --- | --- | --- |
| **S1** | Are you 18 years of age or older? | □ Yes □ No |
| **S2** | Are you currently enrolled as a student at a higher education institution? | □ Yes □ No |
| **S3** | Do you voluntarily agree to participate in this study? | □ Yes □ No |

**If you selected 'No' for any of items S1–S3, please stop here. Thank you for your time.**

# Section A. Background Information

Please select the option that best describes you. Unless otherwise indicated, select only one option for each question.

| **A1 Gender** | □ 1 Male □ 2 Female |
| --- | --- |
| **A2 Age** | □ 1 18–20 years □ 2 21–23 years □ 3 24–26 years □ 4 27 years or older |
| **A3 Level of Study** | □ 1 Junior college □ 2 Undergraduate □ 3 Master's student □ 4 Doctoral student |
| **A4 Academic Field** | □ 1 Humanities and Social Sciences □ 2 Science and Engineering □ 3 Medicine or Agriculture □ 4 Arts or Sports |
| **A5 Living Arrangement** | □ 1 On-campus residence □ 2 Off-campus rental accommodation □ 3 Living with family □ 4 Other |
| **A6 Academic Performance** | □ 1 Poor □ 2 Average □ 3 Good □ 4 Excellent |
| **A7 Academic Stress** | □ 1 Very low □ 2 Low □ 3 High □ 4 Very high |
| **A8 Frequency of Psychological Distress** | □ 1 Never □ 2 Occasionally □ 3 Often □ 4 Almost always |

## B. Teaching Environment (EES)

This section concerns the support provided by teachers, peers, psychological support services, institutional fairness, and feedback channels.

**Response scale: 1 = Strongly disagree, 2 = Disagree, 3 = Neither agree nor disagree, 4 = Agree, and 5 = Strongly agree. For each statement, check the one number that best reflects your actual experience.**

| **Item** | **Statement** | **1** | **2** | **3** | **4** | **5** |
| --- | --- | --- | --- | --- | --- | --- |
| **EES1** | My instructors attend not only to my academic progress but also to my overall well-being. | □ | □ | □ | □ | □ |
| **EES2** | When I experience learning difficulties, instructors provide timely, respectful, and helpful guidance. | □ | □ | □ | □ | □ |
| **EES3** | During collaborative learning, my peers are generally willing to share information, coordinate responsibilities, and provide practical assistance. | □ | □ | □ | □ | □ |
| **EES4** | The psychological counseling or emotional support services provided by my institution are clearly communicated, convenient, and readily accessible. | □ | □ | □ | □ | □ |
| **EES5** | The institution's psychological support services protect privacy and enable students to seek help with confidence. | □ | □ | □ | □ | □ |
| **EES6** | My institution provides courses, lectures, or peer activities that help students understand and maintain their mental health. | □ | □ | □ | □ | □ |
| **EES7** | Rules and requirements affecting students are implemented fairly and consistently. | □ | □ | □ | □ | □ |
| **EES8** | My institution carefully solicits and responds to reasonable student feedback concerning support for mental health and well-being. | □ | □ | □ | □ | □ |

## C. Psychological Safety (PS)

This section concerns how safe you feel when raising questions, expressing divergent views, and trying new approaches in learning interactions.

**Response scale: 1 = Strongly disagree, 2 = Disagree, 3 = Neither agree nor disagree, 4 = Agree, and 5 = Strongly agree. For each statement, check the one number that best reflects your actual experience.**

| **Item** | **Statement** | **1** | **2** | **3** | **4** | **5** |
| --- | --- | --- | --- | --- | --- | --- |
| **PS1** | In class or other learning activities, I can openly ask about things I do not understand. | □ | □ | □ | □ | □ |
| **PS2** | Even if I say something incorrect or give a wrong answer, I am not concerned that I will be ridiculed or belittled. | □ | □ | □ | □ | □ |
| **PS3** | I can actively seek help when I need it without worrying about being evaluated negatively. | □ | □ | □ | □ | □ |
| **PS4** | During class discussions, I am willing to explain my reasoning even when my views are challenged. | □ | □ | □ | □ | □ |
| **PS5** | I am willing to present work that is not yet fully developed in front of others. | □ | □ | □ | □ | □ |

## D. University Belonging (SB)

This section concerns your sense of membership in the school community, acceptance, and emotional connection.

**Response scale: 1 = Strongly disagree, 2 = Disagree, 3 = Neither agree nor disagree, 4 = Agree, and 5 = Strongly agree. For each statement, check the one number that best reflects your actual experience.**

| **Item** | **Statement** | **1** | **2** | **3** | **4** | **5** |
| --- | --- | --- | --- | --- | --- | --- |
| **SB1** | I feel that I am a member of the school community. | □ | □ | □ | □ | □ |
| **SB2** | I feel accepted and respected at my institution. | □ | □ | □ | □ | □ |
| **SB3** | I feel emotionally connected to teachers and fellow students at my institution. | □ | □ | □ | □ | □ |
| **SB4** | After being away from campus for some time, I miss studying and living here. | □ | □ | □ | □ | □ |
| **SB5** | I am willing to participate in and contribute to activities and affairs of the school community. | □ | □ | □ | □ | □ |

## E. Learning Engagement (LE)

This section concerns your energy, attention, effort, persistence, and active participation in learning activities.

**Response scale: 1 = Strongly disagree, 2 = Disagree, 3 = Neither agree nor disagree, 4 = Agree, and 5 = Strongly agree. For each statement, check the one number that best reflects your actual experience.**

| **Item** | **Statement** | **1** | **2** | **3** | **4** | **5** |
| --- | --- | --- | --- | --- | --- | --- |
| **LE1** | I generally feel energetic when engaging in learning activities. | □ | □ | □ | □ | □ |
| **LE2** | I am able to maintain my attention in class and while completing learning tasks. | □ | □ | □ | □ | □ |
| **LE3** | Beyond course requirements, I actively seek additional materials or practice opportunities to deepen my understanding of the content. | □ | □ | □ | □ | □ |
| **LE4** | Even when I encounter difficulties, I persist until I complete my learning tasks. | □ | □ | □ | □ | □ |
| **LE5** | I actively participate in class discussions and other learning activities. | □ | □ | □ | □ | □ |
| **LE6** | I frequently consider how to understand knowledge in greater depth rather than merely completing requirements. | □ | □ | □ | □ | □ |

# F. Psychological Well-Being (PWB)

Please answer with reference to your overall psychological experience during the current semester. The items concern self-acceptance, positive relations, autonomy, environmental mastery, purpose in life, and personal growth.

**Response scale: 1 = Strongly disagree, 2 = Disagree, 3 = Neither agree nor disagree, 4 = Agree, and 5 = Strongly agree. For each statement, check the one number that best reflects your actual experience.**

| **Item** | **Statement** | **1** | **2** | **3** | **4** | **5** |
| --- | --- | --- | --- | --- | --- | --- |
| **PWB1** | I accept myself as I am, including both my strengths and limitations. | □ | □ | □ | □ | □ |
| **PWB2** | When the result of an examination or assignment is disappointing, I do not evaluate myself negatively in every respect. | □ | □ | □ | □ | □ |
| **PWB3** | In important relationships, I feel understood, cared for, and supported. | □ | □ | □ | □ | □ |
| **PWB4** | When choosing courses or planning my development, I make judgments on the basis of my own values and interests. | □ | □ | □ | □ | □ |
| **PWB5** | When faced with expectations from peers or family members, I maintain independent judgment rather than complying uncritically. | □ | □ | □ | □ | □ |
| **PWB6** | I am able to organize my studies and daily life proactively and identify appropriate ways to cope when circumstances change. | □ | □ | □ | □ | □ |
| **PWB7** | I have a clear understanding of the learning and development goals I hope to achieve at this stage. | □ | □ | □ | □ | □ |
| **PWB8** | I can explain how the knowledge and skills I am currently acquiring will support my future development. | □ | □ | □ | □ | □ |
| **PWB9** | I am able to gain new understanding and grow through difficulties or setbacks. | □ | □ | □ | □ | □ |

**This is the end of the questionnaire. Thank you for your thoughtful participation.**
