# Supplementary material for: The predictive association between the teaching environment and psychological wellbeing: psychological safety, university belonging, and learning engagement
Source: Front Psychol. 2026 Oct 20;17:1983869. doi: 10.3389/fpsyg.2026.1983869 (PMC13727545; doi:10.3389/fpsyg.2026.1983869)
Supplement: Supplementary file 1 [file Data_Sheet_1.zip › Supplementary_Material_1_Factor_and_Correlation.docx]

**Factor and Correlation Matrices**

Teaching Environment and Psychological Well-Being Study · *N* = 815

**1. Factor Analysis Matrices**

Based on the randomized split sample, this section reports the complete item-level factor matrices. Table S1 presents the Exploratory Factor Analysis (EFA) results based on Principal Axis Factoring with Promax oblique rotation (*N* = 408). Table S2 reports the standardized factor loadings derived from the Confirmatory Factor Analysis (CFA) maximum-likelihood estimation (*N* = 407).

**Table S1. Complete Exploratory Factor Analysis (EFA) Factor Matrix**

| **Item** | **Factor 1 (EES)** | **Factor 2 (PS)** | **Factor 3 (SB)** | **Factor 4 (LE)** | **Factor 5 (PWB)** | **Communality (h²)** |
| --- | --- | --- | --- | --- | --- | --- |
| EES1 | **0.751** | - | - | - | - | 0.582 |
| EES2 | **0.786** | - | - | 0.102 | - | 0.652 |
| EES3 | **0.734** | - | - | - | - | 0.545 |
| EES4 | **0.768** | - | - | - | - | 0.598 |
| EES5 | **0.771** | - | 0.113 | - | - | 0.620 |
| EES6 | **0.783** | 0.125 | - | - | - | 0.638 |
| EES7 | **0.759** | - | - | - | 0.153 | 0.612 |
| EES8 | **0.742** | - | - | - | - | 0.575 |
| PS1 | - | **0.801** | - | - | - | 0.642 |
| PS2 | 0.104 | **0.844** | - | - | - | 0.720 |
| PS3 | - | **0.767** | - | - | - | 0.583 |
| PS4 | - | **0.706** | 0.134 | - | - | 0.538 |
| PS5 | - | **0.752** | - | - | - | 0.575 |
| SB1 | - | - | **0.754** | - | - | 0.579 |
| SB2 | - | 0.135 | **0.773** | - | - | 0.612 |
| SB3 | - | - | **0.835** | - | - | 0.631 |
| SB4 | 0.120 | - | **0.724** | 0.150 | - | 0.569 |
| SB5 | - | - | **0.748** | - | - | 0.588 |
| LE1 | - | - | 0.131 | **0.778** | - | 0.612 |
| LE2 | - | 0.114 | - | **0.828** | - | 0.657 |
| LE3 | - | - | - | **0.745** | 0.141 | 0.568 |
| LE4 | - | - | - | **0.791** | - | 0.618 |
| LE5 | - | - | - | 0.647 | - | 0.537 |
| LE6 | 0.118 | - | - | **0.785** | - | 0.601 |
| PWB1 | - | - | - | 0.113 | **0.729** | 0.548 |
| PWB2 | - | 0.120 | - | - | **0.784** | 0.605 |
| PWB3 | - | - | - | - | 0.646 | 0.532 |
| PWB4 | - | - | - | - | **0.818** | 0.634 |
| PWB5 | - | - | - | - | **0.772** | 0.589 |
| PWB6 | - | - | - | - | **0.745** | 0.566 |
| PWB7 | 0.142 | - | - | - | **0.718** | 0.551 |
| PWB8 | - | - | 0.124 | - | **0.756** | 0.588 |
| PWB9 | - | - | - | - | **0.730** | 0.552 |

Note: Loadings below 0.15 are suppressed for clarity. Primary loadings are presented in bold.

**Table S2. Complete Confirmatory Factor Analysis (CFA) Standardized Factor Loadings**

| **Construct & Item** | **Standardized Loading (λ)** | **Standard Error (SE)** | **z-value** | **p-value** |
| --- | --- | --- | --- | --- |
| **Teaching Environment (EES)** |  |  |  |  |
| EES1 | 0.772 | 0.057 | 15.17 | < 0.001 |
| EES2 | 0.833 | 0.056 | 18.21 | < 0.001 |
| EES3 | 0.704 | 0.059 | 14.87 | < 0.001 |
| EES4 | 0.807 | 0.058 | 17.53 | < 0.001 |
| EES5 | 0.810 | 0.057 | 17.61 | < 0.001 |
| EES6 | 0.817 | 0.056 | 17.82 | < 0.001 |
| EES7 | 0.789 | 0.057 | 17.06 | < 0.001 |
| EES8 | 0.793 | 0.059 | 17.15 | < 0.001 |
| **Psychological Safety (PS)** |  |  |  |  |
| PS1 | 0.810 | 0.056 | 18.15 | < 0.001 |
| PS2 | 0.838 | 0.053 | 19.33 | < 0.001 |
| PS3 | 0.802 | 0.054 | 18.10 | < 0.001 |
| PS4 | 0.749 | 0.058 | 16.32 | < 0.001 |
| PS5 | 0.770 | 0.056 | 16.92 | < 0.001 |
| **University Belonging (SB)** |  |  |  |  |
| SB1 | 0.782 | 0.060 | 17.13 | < 0.001 |
| SB2 | 0.796 | 0.058 | 17.38 | < 0.001 |
| SB3 | 0.827 | 0.056 | 18.57 | < 0.001 |
| SB4 | 0.702 | 0.061 | 14.73 | < 0.001 |
| SB5 | 0.772 | 0.058 | 16.71 | < 0.001 |
| **Learning Engagement (LE)** |  |  |  |  |
| LE1 | 0.789 | 0.059 | 17.59 | < 0.001 |
| LE2 | 0.829 | 0.057 | 18.91 | < 0.001 |
| LE3 | 0.780 | 0.057 | 17.26 | < 0.001 |
| LE4 | 0.808 | 0.056 | 18.18 | < 0.001 |
| LE5 | 0.750 | 0.060 | 16.27 | < 0.001 |
| LE6 | 0.758 | 0.063 | 16.53 | < 0.001 |
| **Psychological Well-Being (PWB)** |  |  |  |  |
| PWB1 | 0.730 | 0.054 | 15.65 | < 0.001 |
| PWB2 | 0.742 | 0.051 | 16.03 | < 0.001 |
| PWB3 | 0.678 | 0.056 | 14.15 | < 0.001 |
| PWB4 | 0.756 | 0.054 | 16.50 | < 0.001 |
| PWB5 | 0.750 | 0.054 | 16.29 | < 0.001 |
| PWB6 | 0.718 | 0.056 | 15.30 | < 0.001 |
| PWB7 | 0.701 | 0.055 | 14.79 | < 0.001 |
| PWB8 | 0.725 | 0.053 | 15.52 | < 0.001 |
| PWB9 | 0.690 | 0.057 | 14.47 | < 0.001 |

**2. Bivariate Correlation Matrix**

The overall sample data (N = 815) was utilized to estimate the bivariate Pearson correlation coefficients and the corresponding 95% confidence intervals based on Fisher's z-transformation.

**Table S3. Bivariate Correlation Matrix with 95% Confidence Intervals**

| **Variables** | **PS** | **SB** | **LE** | **PWB** |
| --- | --- | --- | --- | --- |
| **EES** | 0.503 [0.450, 0.553] | 0.412 [0.353, 0.468] | 0.335 [0.272, 0.394] | 0.435 [0.378, 0.489] |
| **PS** | - | 0.504 [0.451, 0.553] | 0.418 [0.359, 0.473] | 0.414 [0.356, 0.470] |
| **SB** |  | - | 0.561 [0.512, 0.607] | 0.440 [0.383, 0.494] |
| **LE** |  |  | - | 0.554 [0.504, 0.600] |

Note: EES = Teaching Environment; PS = Psychological Safety; SB = University Belonging; LE = Learning Engagement; PWB = Psychological Well-Being. 95% confidence intervals (CIs) were computed based on the robust Fisher z-transformation. All correlations are significant at the p < 0.001 level.
